# Assessment of the Commitments and Performance of the European Food Industry to Improve Population Nutrition

**DOI:** 10.3389/ijph.2022.1604116

**Published:** 2022-06-01

**Authors:** Iris Van Dam, Emilie Guillon, Ella Robinson, Olivier Allais, Gary Sacks, Stefanie Vandevijvere

**Affiliations:** ^1^ Sciensano, Service of Lifestyle and Chronic Diseases, Brussels, Belgium; ^2^ Université Paris-Saclay, INRAE, UR ALISS, Ivry-sur-Seine, France; ^3^ Alimentation & Santé, UniLaSalle Campus de Beauvais, Beauvais, France; ^4^ Global Obesity Centre (GLOBE), Institute for Health Transformation, Deakin University, Geelong, VIC, Australia

**Keywords:** Europe, food environments, food industry, food supply, nutrient profile, business impact assessment, accountability

## Abstract

**Objectives:** Food companies could play an important role in improving population diets, but often escape accountability through unspecific commitments. This study evaluated nutrition-related commitments and estimated performance of the largest packaged food and non-alcoholic beverage manufacturers, supermarkets and quick-service restaurants (QSR) in Europe.

**Methods:** To quantitatively assess companies’ publicly available commitments in 2020, the “Business Impact Assessment on Obesity and Population Nutrition” was applied. The proportion of sales from ultra-processed and “unhealthy” food categories (product categories not-permitted to be marketed to children) and over time changes in the number of QSR transactions and QSR and supermarket outlets were calculated.

**Results:** Company commitments fell short of best practice recommendations (median overall score of 21%, range: 1%–62%). Food and beverage companies generated 82% (15%–100%) and 58% (1%–100%) sales from ultra-processed and “unhealthy” products, respectively. The number of QSR outlets and transactions substantially increased in Europe since 2011, while QSR commitments to improve population nutrition remained limited.

**Conclusion:** Whilst most companies made some nutrition-related commitments, they did not comply with best practice recommendations. A large proportion of sales was generated from ultra-processed/unhealthy products and QSR outlets increased. Government regulations are urgently needed.

## Introduction

Throughout Europe different food cultures, income levels and inequalities can be observed, but the challenges relating to unhealthy diets and overweight remain largely the same [1]. In 2016, approximately only 41% of the European population was classified as having a normal bodyweight (Body Mass Index, BMI <25 kg/m^2^ and ≥18.5 kg/m^2^) [1, 2]. Genetics may be able to explain weight variations at an individual level, but cannot explain the continued weight gain across populations and age categories [3]. Food environments, defined as “the collective physical, economic, policy and sociocultural surroundings, opportunities and conditions that influence people’s food and beverage choices and nutritional status” [4], are now thought to be the primary drivers of unhealthy diets and obesity [4–7].

Within current food environments food and beverage companies are attempting to profile themselves as responsible actors that are part of the solution to improving population nutrition and reducing obesity, instead of contributing to the underlying problem [8–10]. Solutions proposed by food companies are generally voluntary and self-regulatory in nature [10]. For example, the EU-Pledge is a European wide initiative to address marketing of unhealthy foods and beverages towards children [11, 12]. Although compliance to this pledge by signatory companies is high, this does not translate into effective protection of children from unhealthy food marketing, due to the target audience definition, the limited number of national signatories and the lenient nutrition criteria [13, 14]. An alternative nutrient profiling system to determine whether food products should be permitted to be marketed to children is the World Health Organisation Regional Office for Europe nutrient profile model (WHO-model), which is considerably stricter than the EU-Pledge and allows fewer products to be marketed to children [14, 15].

To ensure that commitments made by the food industry translate into real-world good practices, it is essential to monitor and evaluate them [7]. The Access to Nutrition Index (ATNI) benchmarks the largest food and beverage manufacturers on their nutrition-related policies and practices at a global level [16–20]. The International Network for Food and Obesity/Non-communicable Diseases (NCDs) Research, Monitoring and Action Support (INFORMAS) developed the “Business Impact Assessment on Obesity and Population Nutrition” (BIA-Obesity) based on the ATNI methods, a review of relevant academic papers, WHO documents and other grey literature reports [7, 21]. While the ATNI evaluates commitments and performance of global packaged food and beverage manufacturers to reduce both undernutrition and obesity [16–20], the BIA-Obesity focusses solely on overweight and obesity and is less resource intensive [22]. In addition to packaged food and beverage manufacturers, the BIA-Obesity assessment includes quick-service restaurants (QSR) and supermarkets [7, 21]. Per company the comprehensiveness, transparency, and specificity of commitments and the practices are assessed across six policy domains: “Corporate strategy,” “Product formulation,” “Nutrition labelling,” “Product and brand promotion,” “Product accessibility,” and “Relationships with other organisations.” While for the latter and the first domain the indicators are the same for all food industries (i.e., packaged food and beverage manufacturers, QSR and supermarkets), the indicators within the other four domains differ for QSR and supermarkets as both industries are in direct contact with consumers, something that is rarely the case for packaged food and beverage manufacturers [21]. Collecting company commitments across these policy domains ensures industry accountability, but also makes it possible to assess whether the commitments in place meet best practice examples and as such could be sufficient to improve food environments. Eventually, areas where commitments are currently lacking can be identified [22].

To date, the BIA-Obesity has been applied in six countries [23–28]. This study is the first to apply BIA-Obesity in the European context. This study aimed to quantitatively assess publicly available nutrition-related commitments made by the largest packaged food and beverage manufacturers, supermarkets and QSR in Europe (2020). Company performance was estimated by calculating the proportion of packaged food and beverage sales from ultra-processed and “unhealthy” food categories. For QSR and supermarkets, the number of outlets and annual fast food transactions (the latter for QSR only) were considered, to estimate their presence throughout Europe and link with the importance of having comprehensive, transparent and specific commitments.

## Methods

### Adaptation of the BIA-Obesity Tool and Process to the European Context

The indicators across BIA-Obesity domains relate to company commitments that go beyond legislative requirements. For this reason, before the BIA‐Obesity is applied in a particular jurisdiction, indicators and scoring criteria are modified to suit the particular legislative context.

In collaboration with the INFORMAS team, the BIA-Obesity indicators were adapted to the European context [7, 21]. Firstly, indicators not applicable to the European context were removed, such as those related to the on-pack disclosure of the ingredients list, trans-fat and added sugar content. This is regulated by the European Union (EU) Regulation No 1169/2011 [29].

Secondly, the scoring of the remaining indicators was adapted. Indicators assessing if a commitment was in place were scored higher if the commitment specifically applied to Europe (or referred to more than two European countries) instead of solely being a global commitment. Indicators that scored the content of the commitments, were scored based on the comprehensiveness, transparency, and specificity of the commitment, regardless of whether it was applied at European or global level [21]. If an active declaration was found stating that the company had no activity in a certain area (e.g., committed not to make political donations), the maximum score was assigned. The complete tool, including scoring criteria, can be found in Supplementary File S1.

### Selection of Food Companies

Food companies were selected among four European food industries, namely, packaged food and non-alcoholic beverage manufacturers, QSR and supermarkets. The Euromonitor International Passport database was used to select companies based on their overall market share in both Eastern- and Western Europe per industry in 2017/2018 [30]. Euromonitor uses a geographical definition of Europe, including 17 countries in both Eastern- and Western Europe. Consequently, some non-EU members were also included (Belarus, Georgia, Moldova, Ukraine and Russia for Eastern Europe and Andorra, Iceland, Lichtenstein, Monaco, Norway, Switzerland and Turkey for Western Europe according to the Euromonitor classification).

Selection of packaged food and beverage manufacturers was at company level. For QSR and supermarkets, selection was at brand level (e.g., KFC and Pizza Hut are both brands from Yum! Brands). For QSR, data were available for all 17 West European countries, but only for eight East European countries. Within each industry, the most prominent European companies/brands were selected on two criteria: 1) ≥1% market share in Eastern- and Western Europe, 2) Presence across East- and West European countries. For example, companies only present within the aforementioned non-EU countries, were excluded.

For packaged food manufactures an additional selection was conducted based on companies’ contribution to the sales of specific food categories such as “Breakfast cereals,” “Confectionery,” “Ice-cream and frozen desserts,” “Sweet biscuits and cereal bars,” “Drinking milk products,” “Yoghurts,” “Savoury snacks” and “Ready meals.” For the purpose of this project, alcoholic beverages, edible oils, bottled water, infant formula and baby foods were excluded.

### Data Collection

#### Nutrition-Related Commitments

An internet search was conducted for each selected company to identify publicly available nutrition-related commitments [7]. The available data were downloaded or screenshots were taken. Where it existed, the European company website was searched alongside the global website. Brand websites were also included. For supermarkets, an additional selection of national company websites was searched to identify commitments made in two or more individual European countries. Due to language barriers these national websites were limited to websites in English, Dutch, French, Spanish and German. Where available, financial and corporate social responsibility reports were also examined. Lastly, industry pledges and initiatives (i.e., the EU-Pledge and IFBA reformulation commitments) were taken into account.

As BIA-Obesity indicators are identical for packaged food and beverage manufacturers and several companies are active within both areas, both industries are discussed together throughout the article.

#### Performance Estimation Metrics

Due to limited data available at European level to assess performance as recommended by INFORMAS, performance was estimated using Euromonitor International sales data (2018) [7, 30]. Food companies were not contacted with the request to share nutritional data.

For packaged food and beverage manufacturers, the healthiness of product sales was used as a measure to assess company “performance” in two BIA-Obesity domains: “Product formulation” and “Product and brand promotion.”

Data on product categories sold by each company were collected for 27 European countries, 13 in Eastern- and 14 in Western Europe. The healthiness of these product categories was assessed using two classification systems, the NOVA-classification and the WHO-model [15, 30, 31]. The NOVA-classification categorises products into four groups according to the level of processing: 1) Unprocessed or minimally processed foods, 2) Processed culinary ingredients, 3) Processed foods and 4) Ultra-processed foods [31], and was used in this study to calculate, for each selected company and across European countries, the proportion of packaged food/beverage sales from ultra-processed products. The WHO-model is used to determine whether products are permitted to be marketed to children. While some product categories are entirely permitted or not-permitted to be marketed to children, for some product categories, nutrient thresholds are defined. Once a product exceeds the threshold for one nutrient, it is no longer permitted to be marketed to children. In addition to the WHO-model categories that are entirely not-permitted to be marketed to children (category 1, 2, 4a, 4c and 5), also “Milk drinks with sugar” (part of category 4b) and “Sweetened soft drinks” (part of category 4d) were considered as not-permitted [15]. An overview of the different WHO-model categories and how they were classified at category level for the purpose of this study can be found in Supplementary File S2. An overview on how Euromonitor food categories were classified according to both the NOVA and the WHO-model classification can be found in Supplementary File S3.

For QSR and supermarkets, the number of outlets and annual fast food transactions (the latter for QSR only) was obtained from Euromonitor, to estimate their presence throughout Europe and link with the importance of having strong commitments, especially within the “Product accessibility” domain. The number of QSR outlets and transactions for McDonald’s only included the brand McDonald’s (not McCafé) and for Pizza Hut only included Pizza Hut (not Pizza Hut Express). Similarly, the number of outlets for Auchan did not comprise Auchan City or Auchan outlets in hands of CONAD, Carrefour outlets did not comprise Carrefour Express, Carrefour Market or Carrefour Planet and Tesco outlets did not comprise Tesco Express and Tesco Extra.

### Data Analysis

#### Nutrition-Related Commitments

The scoring of the commitments was completed in Microsoft Excel. Supplementary File S4 provides an example of how the commitments were scored. The scores were assigned by two authors (EG and IVD) and subsequently a sample of six companies (two companies per food industry) were re-scored blindly by a third author (ER). Scoring discrepancies were discussed until an agreement was obtained. The scores per domain and food sector were weighted according to the BIA-Obesity methodology (Supplementary File S5) [21].

The median scores (range) for the commitments per BIA-Obesity domain were calculated for each food industry and across food industries.

#### Performance Estimation Metrics

The proportion (range, standard deviation (SD)) of sales for ultra-processed and not-permitted food categories (i.e., “unhealthy” food categories), as well as the average number of QSR outlets and annual fast food transactions in 2018, were calculated per company across European countries. To estimate changes over time, the average percent change was calculated over a 10-year period (2009–2018) for packaged food and beverage manufacturers and over an 8-year period (2011–2018) for supermarkets and QSR (due to Euromonitor data availability).

## Results

A total of 30 companies were assessed, 17 packaged food and beverage manufacturers, six QSR and seven supermarkets. An overview of the included companies together with their market shares in Eastern- and Western Europe and the number of countries they were present with ≥1% market share can be found in Table 1.

**TABLE 1:** Companies included for the Business Impact Assessment on Obesity and Population Level Nutrition (BIA-Obesity) in Europe, 2020, together with their market share or brand share in Eastern- and Western Europe and the number of countries they operate in. Sourced from Euromonitor 2017/18. Assessment of the commitments and performance of the European food industry to improve population nutrition, Europe, 2020.

Company	Market share 2017/2018 (%)		Number of countries operating in with ≥1% market share	
	Eastern Europe	Western Europe	Eastern Europe	Western Europe
**Packaged Food Manufacturers**				
Danone Group	3	2	10/17	9/17
Ferrero Group	2	2	12/17	8/17
Intersnack Knabber-Gebäck GmbH & Co KG^1^	0.3	0.5	0/17	2/17
Kellogg Co^a^	0.3	0.6	0/17	1/17
Lactalis, Groupe	1	2	7/17	7/17
Mars Inc.	2	1	16/17	10/17
Mondelēz International Inc.	2	2	14/17	15/17
Nestlé SA	2	2	13/17	11/17
Oetker-Gruppe^a^	0.2	0.5	0/17	1/17
Pepsico Inc.^b^	3	0.9	6/17	7/17
Unilever Group	1	2	12/17	16/17
Total Market Share 2018	17	15		
**Non-Alcoholic Beverage Manufacturers**				
Britvic Plc	—	2	—	3/17
Coca-Cola Co	18	21	17/17	17/17
Eckes-Granini Group GmbH	0.6	2	3/17	5/17
Maspex Wadowice Grupa	3	—	7/17	—
Pepsico Inc.^b^	12	6	17/17	17/17
Red Bull GmbH	2	3	0/17	5/17
Suntory Ltd.	0.2	3	1/17	7/17
Total Market Share 2018	23	33		
**Quick-Service Restaurants^c^**				
Burger King (Restaurant Brands International Inc.)	8	5	7/8	16/17
Domino’s Pizza Inc.	0.8	2	6/8	16/17
KFC (Yum! Brands Inc.)	12	3	8/8	10/17
McDonald’s (McDonald’s Corp)	27	19	8/8	17/17
Pizza Hut	1	1	6/8	13/17
Subway (Doctor’s Associates Inc.)	2	2	7/8	11/17
Total Brand Share 2017	51	31		
**Supermarkets^d^**				
Aldi	0.4	5	1/17	9/17
Auchan (Auchan Group)	2	2	5/17	2/17
Carrefour (Carrefour SA)	0.7	3	3/17	5/17
Lidl (Schwarz Beteiligungs GmbH)	4	5	9/17	15/17
Maxima (Vilniaus Prekyba UAB)^e^	0.8	—	3/17	—
Spar (Internationale Spar Centrale BV)	1	1	5/17	7/17^f^
Tesco (Tesco Plc)	2	2	4/17	2/17
Total Brand Share 2018	10	17		

a Added based on their importance towards addressing obesity in general and among children, as determined by their contribution to the sales of specific Euromonitor food categories such as “Breakfast cereals,” “Confectionery,” “Ice-cream and frozen desserts,” “Sweet biscuits and cereal bars,” “Drinking milk products,” “Yoghurts,” “Savoury snacks” and “Ready meals.” Intersnack Knabber-Gebäck GmbH & Co KG did not have more than 1% market share in Eastern and Western Europe, but was a considerable contributor to the sales of “Savoury snacks” with 5.3% and 9.1% of the market share of “Savoury snacks” in Eastern and Western Europe, respectively. Kellogg Co in turn was the biggest company selling “Breakfast cereals” in both Eastern and Western Europe with a market share of 6.6% and 27%, respectively, within this food category. They also substantially contributed to the sales of “Sweet biscuits and cereal bars” and “Savoury snacks,” making them important to include towards addressing childhood obesity. Lastly, Oetker-Gruppe was identified as the biggest company specialised in ‘Ready meals’ in Western Europe with a market share of 5.5% and was also among the top 5 in Eastern Europe with a market share of 2.3%.

b Pepsico Inc was included both as packaged food and beverage manufacturer. This was not done for other companies already included as packaged food manufacturers, such as Danone and Nestlé, as they, although having a high market share for beverages, showed to mainly contribute to the sales of bottled water and derivate products such as sugared/juicy/aromatic waters.

c Brand share was defined as the brand share among “Chained consumer food services” as obtained from Euromonitor 2017/2018. Euromonitor defines “Chained Consumer Foodservices” as: “Chained units are defined by 10 or more units. An exception is made for international chains that have a presence of fewer than 10 units in a country. In this case, they are still considered to be chained units.”

d Brand share was defined as the brand share among “Grocery Retailers,” defined as: “Retailers selling predominantly food/beverages/tobacco and other everyday groceries. This is the aggregation of hypermarkets, supermarkets, discounters, convenience stores, independent small grocers, chained forecourt retailers, independent forecourt retailers, food/drink/tobacco specialists and other grocery retailers.” by Euromonitor 2017/2018.

e Maxima (Vilniaus Prekyba UAB) was added to the selection as they were the biggest supermarket in Estonia, Latvia and Lithuania with a market share of 17.5%, 24.5% and 32.8%, respectively. The only other supermarkets present in this geographical area was Lidl in Lithuania.

f Spar (Internationale Spar Centrale BV) had an additional market share of 0.9% in two West European countries bringing the overall coverage to nearly 9/17.

**FIGURE 1:**
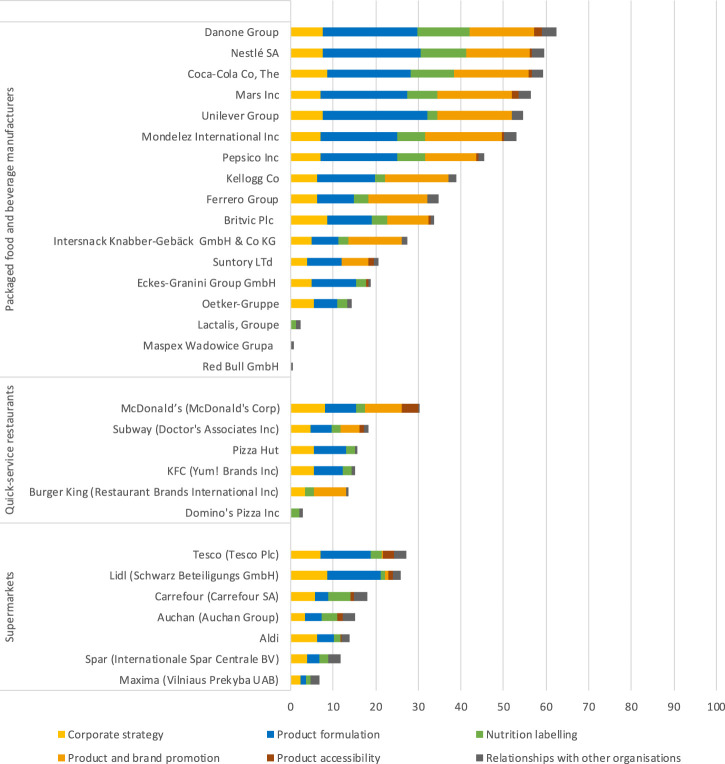
Overall Business Impact Assessment on Obesity and Population Level Nutrition (BIA-Obesity) scores for selected packaged food and beverage manufacturers, quick-service restaurants and supermarkets in Europe, 2020. Assessment of the commitments and performance of the European food industry to improve population nutrition, Europe, 2020.

The overall BIA-Obesity score ranged from 1% (Maspex Wadowice and Red Bull GmbH) to 62% (Danone), with a median score across all companies of 21%. The median scores for packaged food and beverage manufacturers, QSR and supermarkets were 35% (range: 1%–62%), 15% (range: 3%–30%) and 15% (range: 7%–27%), respectively (Figure 1; Table 2).

**TABLE 2:** The total Business Impact Assessment on Obesity and Population Level Nutrition (BIA-Obesity) scores as well as the scores for the individual domains per company (based on publicly available data, 2020). Assessment of the commitments and performance of the European food industry to improve population nutrition, Europe, 2020.

Company name	Total BIA-score (%)	Corporate strategy (%)	Product formulation (%)	Nutrition labelling (%)	Product and brand promotion (%)	Product accessibility (%)	Relationships with other organisations (%)
**Packaged Food Manufacturers**							
Danone Group	62	77	74	62	50	38	67
Ferrero Group	35	62	29	18	46	0	50
Intersnack Knabber-Gebäck GmbH & Co KG	27	48	21	12	42	2	28
Kellogg Co	39	63	45	12	50	0	39
Lactalis, Groupe	2	0	0	6	0	0	22
Mars Inc.	56	70	68	35	58	27	61
Mondelez International Inc.	53	70	61	32	60	10	56
Nestlé SA	59	77	76	53	50	13	56
Oetker-Gruppe	14	55	18	12	0	0	17
Pepsico Inc.*	46	70	61	32	40	10	28
Unilever Group	55	77	82	12	58	2	50
Median	46	70	61	18	50	2	50
Min	2	0	0	6	0	0	17
Max	62	77	82	62	60	38	67
**Non-Alcoholic Beverage Manufacturers**							
Britvic Plc	34	87	35	18	33	10	17
Coca-Cola Co, The	59	87	65	50	58	17	56
Eckes-Granini Group GmbH	19	50	35	12	0	8	11
Maspex Wadowice Grupa	1	0	0	0	0	0	17
Pepsico Inc.*	46	70	61	32	40	10	28
Red Bull GmbH	1	0	0	0	0	0	11
Suntory Ltd.	21	40	27	0	21	25	22
Median	21	50	35	12	21	10	17
Min	1	0	0	0	0	0	11
Max	59	87	65	50	58	25	56
Median overall (packaged food & non-alcoholic beverage manufacturers)	35	63	35	12	42	8	28
Min overall (packaged food & non-alcoholic beverage manufacturers)	1	0	0	0	0	0	11
Max overall (packaged food & non-alcoholic beverage manufacturers)	62	87	82	62	60	38	67
**Quick-Service Restaurants**							
Burger King (Restaurant Brands International Inc.)	14	33	0	14	31	0	9
Domino’s Pizza Inc.	3	0	0	14	0	0	14
KFC (Yum! Brands Inc.)	15	55	28	14	0	0	14
McDonald’s (McDonald’s Corp)	30	80	30	14	35	18	5
Pizza Hut	16	55	30	14	0	0	14
Subway (Doctor’s Associates Inc.)	18	47	20	14	18	5	23
Median	15	51	24	14	9	0	14
Min	3	0	0	14	0	0	5
Max	30	80	30	14	35	18	23
**Supermarkets**							
Aldi	14	63	16	9	0	2	33
Auchan (Auchan Group)	15	33	16	24	0	6	61
Carrefour (Carrefour SA)	18	57	13	33	0	4	67
Lidl (Schwarz Beteiligungs GmbH)	26	87	50	7	3	4	39
Maxima (Vilniaus Prekyba UAB)	7	23	5	7	0	0	39
Spar (Internationale Spar Centrale BV)	12	40	11	15	0	0	56
Tesco (Tesco Plc)	27	70	47	17	2	13	56
Median	15	57	16	15	0	4	56
Min	7	23	5	7	0	0	33
Max	27	87	50	33	3	13	67
Overall median	21	57	29	14	18	4	28
Overall min	1	0	0	0	0	0	5
Overall max	62	87	82	62	60	38	67

*Pepsico was assessed as both a packaged food as well as non-alcoholic beverage manufacturer.

The best performing companies within the “Corporate strategy” domain made specific, measurable, achievable, relevant and time bound (SMART) targets within their overarching nutrition strategy, referred to global priorities (WHO recommendations and Sustainable Development Goals) and published regular reports on their approach to population nutrition. Within the “Product formulation” domain, best performing companies committed to not use artificial trans-fat and had some SMART targets in place to reduce either salt, saturated fats, sugar and energy content of products. Within the “Nutrition labelling” domain, best performing companies provided nutritional information online on a per 100 g/ml basis while supporting a European wide implementation of the Nutri-Score and linking the use of nutrition and health claims with the nutritional profile of products. Companies scoring well within the “Product and brand promotion” domain were a signatory to the EU-Pledge and made some additional commitments to not sponsor or market in settings where children gather using unhealthy products. Only limited commitments were found within the “Product accessibility” domain with best performing companies committing to increase the proportion of healthy products within their portfolio as well as supporting some forms of taxation to make healthier foods relatively cheaper and unhealthy foods relatively more expensive. The latter domain is especially important for QSR and supermarkets. Best performing QSR committed to not provide free refills for soft drinks and provided healthy drink and side items within combination meals while best performing supermarkets committed for checkouts to be free from unhealthy items. Within the last domain, “Relationships with other organisations,” best performing companies disclosed supported professional organisations, external research, nutrition education and active lifestyle programs and involvement in public-private partnerships as well as committed to not make political donations.

### Packaged Food and Non-Alcoholic Beverage Manufacturers

The domain “Corporate strategy” scored the highest with a median score of 63% (range: 0%–87%). The domain “Product accessibility” obtained the lowest score, with a median score of 8% (range: 0%–38%).

Packaged food manufacturers that obtained an overall score above 50% were Danone (62%), Nestlé (59%), Mars (56%) and Unilever (55%). Among beverage manufacturers Coca-Cola obtained the highest overall BIA-score (59%), followed by PepsiCo (46%), Britvic (34%) and the Eckes-Granini Group (19%) (Figure 1; Table 2).

Within the domain “Product formulation,” 14 out of the 17 selected packaged food and beverage manufactures had some commitments, with a median score of 35% (range: 0%–82%). Packaged food manufacturers scored considerably higher than beverage manufacturers, with a median score of 61% (range: 0%–82%), compared to 35% (range: 0%–65%) (Table 2).

**FIGURE 2:**
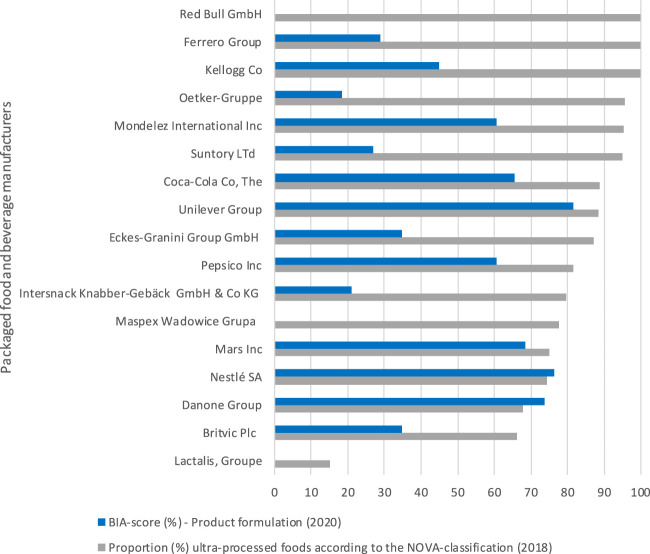
The Business Impact Assessment on Obesity and Population Level Nutrition score (BIA-score) for the domain “Product formulation” (%) compared with the proportion of sales coming from food groups that are ultra-processed (according to NOVA in 2018) per selected packaged food and beverage manufacturer. Assessment of the commitments and performance of the European food industry to improve population nutrition, Europe, 2020.

Packaged food and beverage manufactures generated on average 82% (range: 15%–100%) of sales from ultra-processed foods, or 79% (range: 15%–100%) and 85% (range: 66%–100%), respectively. Apart from Lactalis, that generated only 15% of sales from ultra-processed foods, there were no companies that generated less than 65% of sales from ultra-processed foods. Among the 17 selected packaged food and beverage manufactures, sales generated by ultra-processed foods on average increased over the last 10 years (2009–2018) for six of the companies (+4%, range: 0.9%–9%), did not change for two and decreased for nine (−7%, range: −0.2% to −15%) (Table 3). As shown in Figure 2, companies with stronger commitments in the domain of “Product formulation” did not have healthier product portfolio’s according to the sales generated from ultra-processed foods compared to those with weaker commitments within this domain.

**TABLE 3:** The performance indicators per company and food industry (packaged food and beverage manufacturers^a^, quick-service restaurants^b^, supermarkets^c^). Assessment of the commitments and performance of the European food industry to improve population nutrition, Europe, 2020.

Company name	Performance indicators					
	Proportion (%) of sales not-permitted to be marketed to children across Europe according to WHO-model (2018)			Proportion (%) of sales that are ultra-processed across Europe according to the NOVA-classification (2018)		
	Average (Min – Max)	Standard deviation	% Change (2009–2018)	Average (Min – Max)	Standard deviation	% Change (2009–2018)
**Packaged Food Manufacturers**						
Danone Group	13 (0–71)	16	12.8	68 (37–98)	19	5.2
Ferrero Group	79 (0–100)	35	0.3	100 (100–100)	0	0.0
Intersnack Knabber-Gebäck GmbH & Co KG	1 (0–12)	3	79.2	79 (0–100)	23	−0.4
Kellogg Co	27 (0–64)	16	−22.5	100 (97–100)	1	−0.2
Lactalis, Groupe	6 (0–20)	7	−11.8	15 (0–47)	15	−11.2
Mars Inc.	69 (0–100)	35	−0.4	75 (0–100)	37	0.0
Mondelēz International Inc.	83 (0–100)	22	3.9	95 (0–100)	20	4.6
Nestlé SA	48 (0–94)	28	−17.1	74 (0–100)	30	−11.2
Oetker-Gruppe	39 (0–100)	33	17.5	96 (39–100)	13	9.4
Pepsico Inc.*	60 (0–100)	31	−15.6	82 (0–100)	28	−7.1
Unilever Group	52 (0–74)	16	8.7	89 (0–100)	23	2.1
Average	43 (1–83)			79 (15–100)		
Standard Deviation	27			23		
**Beverage Manufacturers**						
Britvic Plc	67 (0–100)	47	−13.7	66 (0–100)	46	−14.8
Coca-Cola Co, The	91 (0–100)	19	−4.2	89 (0–100)	19	−6.0
Eckes-Granini Group GmbH	95 (0–100)	22	−4.8	87 (0–100)	30	−5.8
Maspex Wadowice Grupa	61 (0–100)	43	2.2	78 (0–100)	38	0.9
Pepsico Inc.*	60 (0–100)	31	−15.6	82 (0–100)	28	−7.1
Red Bull GmbH	100 (100–100)	0	3.9	100 (100–100)	0	3.9
Suntory Ltd.	95 (0–100)	21	−4.6	95 (0–100)	21	−4.4
Average	81 (60–100)			85 (66–100)		
Standard Deviation	16			10		
Average packaged food & beverage manufacturers	58 (1–100)			82 (15–100)		
Standard Deviation packaged food & beverage manufacturers	31			20		
	**Number of outlets across Europe (2018)**			**Number of annual fast food transactions across Europe (2018)**		
	**Total Outlets**		**% Change (2011–2018)**	**Total transactions (x1000)**		**% Change (2011–2018)**
**Quick-Service Restaurants**						
Burger King (Restaurant Brands International Inc.)	4608		75.8	919128		92.0
Domino’s Pizza Inc.	3523		132.7	160300		188.4
KFC (Yum! Brands Inc.)	3102		127.1	527613		132.1
McDonald’s (McDonald’s Corp)	8714		19.1	3311362		23.2
Pizza Hut	1477		24.0	61676		33.0
Subway (Doctor’s Associates Inc.)	5542		69.3	267542		59.4
Average	4494		75	874603		88
Min	1477		19	61676		23
Max	8714		133	3311362		188
**Supermarkets**						
Aldi	7992		6.6			
Auchan (Auchan Group)	764		238.1			
Carrefour (Carrefour SA)	1721		78.3			
Lidl (Schwarz Beteiligungs GmbH)	10581		9.4			
Maxima (Vilniaus Prekyba UAB)	479		14.9			
Spar (Internationale Spar Centrale BV)	8551		6.6			
Tesco (Tesco Plc)	1358		−1.5			
Average	4492		50			
Min	479		−2			
Max	10581		238			

a For packaged food and beverage manufactures the proportion of sales coming from food groups not-permitted to be marketed to children (according to the World Health Organisation, WHO) and ultra-processed (according to NOVA) in 2018 is provided, including the change over the past 10 years (2009–2018).

b For quick-service restaurants (QSR) the number of outlets and annual fast food transactions as well as the change over time is provided (2011–2018). Rather than reflecting QRS performance, these data were retained to highlight the importance for QSR to make strong commitments.

c For supermarkets the number of outlets and change over time is provided (2011–2018).

*Pepsico was assessed as both a packaged food as well as non-alcoholic beverage manufacturer.

**FIGURE 3:**
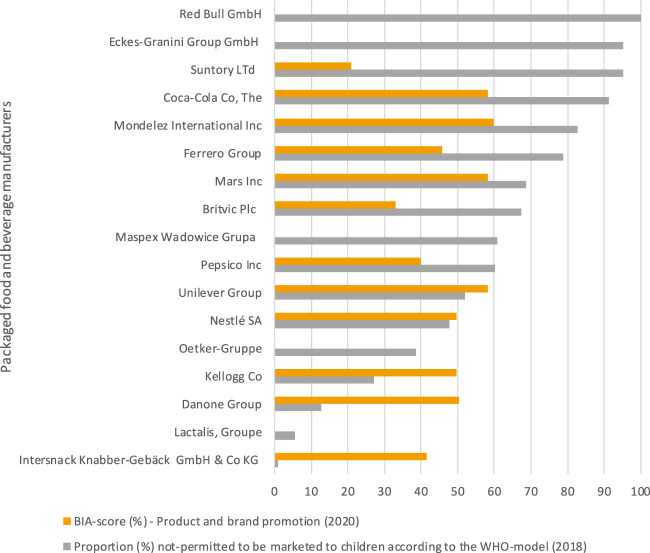
The Business Impact Assessment on Obesity and Population Level Nutrition score (BIA-score) for the domain “Product and brand promotion” (%) compared with the proportion of sales coming from food groups that are not-permitted to be marketed to children (according to the World Health Organisation Regional Office for Europe Nutrient Profile model, WHO-model in 2018) per selected packaged food and beverage manufacturer. Assessment of the commitments and performance of the European food industry to improve population nutrition, Europe, 2020.

Similar to the domain “Product formulation,” 14 out of the 17 selected packaged food and beverage manufactures committed to limit advertising to children below 12-year of age, with the domain “Product and brand promotion” obtaining a median score of 42% (range: 0%–60%). Category specific sales data however revealed that selected packaged food and beverage manufacturers generated on average 58% (range: 1%–100%) of their 2018 sales across Europe from “unhealthy” food categories. Beverage manufactures generated almost all of their sales (average: 81%, range: 60%–100%) from these food categories, whilst for packaged food manufacturers this was approximately half of all sales (average: 43%, range: 1%–83%). Over a 10-year period (2009–2018), eight companies had on average increased sales (+16%, range: 0.3%–79%) from “unhealthy” food categories, whilst this decreased for the remaining nine companies (−11%, range: −0.4% to −23%) (Table 3). In line with the findings in the domain “Product formulation” and shown in Figure 3, companies with stronger commitments in the domain of “Product and brand promotion” did not have healthier product portfolio’s according to the sales generated from “unhealthy” food categories compared to those with weaker commitments in these domains.

### Quick-Service Restaurants

Similar to packaged food and beverage manufacturers, the domain “Corporate strategy” was the highest scoring and “Product accessibility” the lowest scoring domain, with median scores of 51% (range: 0%–80%) and 0% (range: 0%–18%), respectively (Figure 1; Table 2).

McDonald’s obtained the highest overall BIA-Obesity score (30%) as well as the highest score in all domains except for the “Relationships with other organisations” domain, where the highest score was obtained by Subway (23%). Subway, Pizza Hut, KFC and Burger King, all obtained overall scores between 14% and 18%. Domino’s Pizza had the lowest overall BIA-Obesity score (3%).

The limited nutrition-related commitments made by QSR, reflected in a median overall BIA-score of 15% (range: 3%–30%), may be of concern as the selected QSR on average counted 4494 European outlets (range: 1477–8714) and 875 million annual fast food transactions (range: 62 million-3311 million) across Europe in 2018. Both the number of outlets and annual transactions substantially increased since 2011 with on average 75% (range: 19%–133%) and 88% (range: 23%–188%), respectively (Table 3).

### Supermarkets

As with the other sectors, the domain “Corporate strategy” was the highest scoring domain with a median score of 57% (range: 23%–87%). Unlike other sectors, the lowest scoring domain was “Product and brand promotion,” with a median score of 0% (range: 0%–3%) (Figure 1; Table 2).

Tesco obtained the highest overall BIA-Obesity score (27%), closely followed by Lidl (26%). Across the individual domains, Lidl scored the highest within “Corporate strategy” (87%), “Product formulation” (50%) and “Product and brand promotion” (3%). Carrefour scored the highest within “Nutrition labelling” (33%) and “Relationships with other organisations” (67%) and Tesco within “Product accessibility” (13%).

The selected supermarkets on average counted 4492 outlets across Europe in 2018 (range: 479–10,581). The number of outlets increased since 2011 for all supermarkets, apart from Tesco, with on average 50% (range: −2%–238%) (Table 3).

## Discussion

BIA-Obesity scores showed that most selected packaged food and beverage manufacturers, QSR and supermarkets recognised their role in improving food environments, but fell short of recommended best practices. Publicly available nutrition-related commitments largely differed in levels of transparency, specificity and comprehensiveness, with overall scores ranging from 1% to 62%.

The median overall BIA-Obesity score across food industries in Europe was lower than what was found in Australia and New Zealand (21% vs. 41% and 38%, respectively). Previous studies showed that scores typically increase for companies engaging with the BIA-Obesity [24, 26, 28]. As such the difference in scores is likely due to the European assessment being based on only publicly available data, whereas for Australia and New Zealand the assessment included internal policy information provided by companies [26, 28]. Regardless of the root of the lower BIA-Obesity score, the lack of comprehensiveness, specificity and transparency of the publicly available commitments is concerning in light of their influence on food environments [10, 32–34].

“Corporate strategy” was the highest scoring domain, emphasizing that companies like to profile themselves as part of the solution to reducing obesity and improving population nutrition [8–10]. “Product accessibility” was the lowest scoring domain. The low scores within the “Accessibility” domain could potentially be explained by the pricing and distribution of healthier products being less of a concern for companies or being more complex due to the number of actors involved [18, 19, 28]. These findings are similar to previous findings [24, 26, 28] and are also in line with findings from the ATNI 2018 Global Index, which identified “Governance” as the highest scoring and “Accessibility” the lowest scoring domain [19].

Companies could strengthen their role in improving food environments through the enhancement of their nutrition-related commitments. To meet best practice recommendations they could develop SMART targets for product reformulation using an official nutrient profiling system, commit to only label products with nutrition and health claims when products are healthy and develop a marketing policy that applies to children up to the age of 18 (applying the WHO-model). QSR could commit to only advertise “healthy” sides and drinks in combination meals, commit to not use price incentives such as supersizing and commit to not open new stores near schools. Supermarkets could commit to limit multi-buy specials on unhealthy foods, dedicate a maximum amount of shelf/floor space to less healthy products and limit the placement of unhealthy items at high-traffic areas [21]. Such commitments and practices could help the food industry to move beyond profiling themselves as responsible actors [8–10] towards actively improving the healthiness of food environments and population diets [35, 36].

No associations were observed between commitment scores and performance estimation metrics for packaged food and beverage manufactures. Across Europe in 2018 on average 82% and 58% of sales were generated from ultra-processed and “unhealthy” food categories, respectively. These findings indicate that companies with stronger reformulation and marketing to children commitments are still deriving a large proportion of their sales from ultra-processed and unhealthy products. The high proportion of sales derived from ultra-processed foods is particularly concerning within the growing body of literature showing an association between the consumption of ultra-processed foods and overweight [37–39]. The sales generated from “unhealthy” foods are likely an underestimation, as the study only classified products that are not-permitted to be marketed to children under any circumstances. Foods and beverages that are within other WHO-model categories may still exceed the predefined nutrient-thresholds and in practice be not-permitted to be marketed to children [15].

For QSR, scores for commitments were low, while the number of outlets and annual fast food transactions increased substantially over the last 8 years. Although market expansion and thus an increase in the number of outlets and fast food transactions is an inherit aim of the food industry [10, 40], this may be concerning as the increase in annual fast food transactions as well as the proximity of QSR outlets to schools and homes have been positively associated with a BMI increase [34, 41]. Likewise, countries that implemented stricter policies to regulate fast food consumption also experienced a slower increase in BMI [41, 42]. Nonetheless, more research using European-wide nutritional data from QSR is required to assess whether (un)healthy products are responsible for the observed increase in annual fast food transactions.

Policy measures already in place at European level are the obligatory on-pack nutritional information and trans-fat regulation [43, 44]. Across individual European countries, policies have been implemented to support healthy nutrition and physical activity within the school environment, support self-regulatory marketing and reformulation initiatives and a growing support for front-of-pack labelling [1, 45]. Nevertheless, European countries are not on track to meet global nutrition-related targets [1]. These findings, combined with our results that show that food industry nutrition-related commitments fall short of best practice recommendations, highlight the need for more ambitious government regulations, both at European level and across countries.

This study has several strengths. It was the first to evaluate the comprehensiveness, specificity and transparency of publicly available nutrition-related commitments in the European context applying the BIA-Obesity tool. It pointed out domains where commitments were in place to improve food environments and highlighted areas for improvement. By estimating performance it also emphasized the need to improve the relative availability of healthier food choices across Europe while decreasing the proportion sales generated from ultra-processed and unhealthy products. Nonetheless, several limitations were identified. This study solely included publicly available information and as such was not designed to capture internal company commitments. A clear distinction between companies was however evident. Additionally, information was primarily obtained from global company websites and reports. As a result, it was not always clear how commitments were applied in Europe or within individual European countries. For supermarkets in particular, European and global level information was limited and difficult to obtain as the majority of supermarkets operated at the country level. Lastly, due to limited data available at European level, performance across food industries could only be estimated within a few BIA-Obesity domains.

To overcome aforementioned limitations, future research should apply the BIA-Obesity within individual European countries, especially for supermarkets, and data on the nutritional composition of product portfolios, labelling practices, the availability/affordability of products and promotion to children should be collected to more accurately assess performance across all domains of BIA-Obesity. Following the findings and recommendations of this study, the authors applied the BIA-Obesity tool and process in both Belgium and France. Both studies included detailed performance metrics which enabled a more accurate assessment of the relationship between company commitment BIA-Obesity scores and practices [46, 47].

In conclusion, this study found that most major European packaged food and beverage manufacturers, QSR and supermarkets made commitments to improve food environments, albeit with varying transparency, specificity and comprehensiveness. These commitments did not meet best practice recommendations. Even though food companies recognised their role in improving food environments and profiled themselves as part of the solution, the relative availability of healthier packaged food and beverage choices was limited across Europe. As a result, more ambitious government regulations are needed, both at European- and national-level.

## Data Availability

The datasets used and/or analysed during the current study are available from the corresponding author on reasonable request.

## Abbreviations

ATNI, access to nutrition index; BIA-Obesity, business impact assessment on obesity and population nutrition; BMI, body mass index; EU, European Union; IFBA, International Food and Beverage Alliance; INFORMAS, The International Network for Food and Obesity/Non-communicable Diseases (NCDs) Research, Monitoring and Action Support; NCD, non-communicable disease; QSR, Quick-Service Restaurant(s); SMART, specific, measurable, achievable, relevant and time bound; WHO, World Health Organisation; WHO-model, World Health Organisation Regional Office for Europe nutrient profile model.
