# Supplementary material for: Assessment of the Commitments and Performance of the European Food Industry to Improve Population Nutrition
Source: Int J Public Health. 2022 Jun 1;67:1604116. doi: 10.3389/ijph.2022.1604116 (PMC9198223; doi:10.3389/ijph.2022.1604116)
Supplement: Supplementary file 1 [file DataSheet2.docx]

Supplementary file 2: The World Health Organisation Regional Office for Europe Nutrient Profile model (WHO-model) categories and how the classification was applied at category level. *Assessment of the commitments and performance of the European food industry to improve population nutrition, Europe, 2020.*

| **Group** | **Name** | **Marketing to children** |
| --- | --- | --- |
| **1** | Chocolate and sugar confectionery, energy bars, and sweet toppings and desserts | Not-permitted |
| **2** | Cakes, sweet biscuits and pastries; other sweet bakery wares, and dry mixes for making such | Not-permitted |
| **3** | Savoury snacks | Potentially permitted |
| **4** | Beverages |  |
| **4A** | a) Juices | Not-permitted |
| **4B** | b) Milk drinks | With sugar: Not-permitted, Others: Potentially permitted |
| **4C** | c) Energy drinks (often contain o.a. guarana, taurine, glucuronolactone and vitamins) | Not-permitted |
| **4D** | d) Other beverages (Soft drinks, sweetend beverages) | Sweetened soft drinks: Not-permitted, Others: Potentially permitted |
| **5** | Edible ices | Not-permitted |
| **6** | Breakfast cereals | Potentially permitted |
| **7** | Yoghurts, sour milk, cream and other similar foods | Potentially permitted |
| **8** | Cheese | Potentially permitted |
| **9** | Ready-made and convenience foods and composite dishes | Potentially permitted |
| **10** | Butter and other fats and oils | Potentially permitted |
| **11** | Bread, bread products and crisp breads | Potentially permitted |
| **12** | Fresh or dried pasta, rice and grains | Potentially permitted |
| **13** | Fresh and frozen meat, poultry, fish and similar +eggs | Permitted |
| **14** | Processed meat, poultry, fish and similar | Potentially permitted |
| **15** | Fresh and frozen fruit, vegetables and legumes | Permitted |
| **16** | Processed fruit, vegetables and legumes | Potentially permitted |
| **17** | Sauces, dips and dressings | Potentially permitted |

Supplementary file 3: Euromonitor food groups (2019) and their respective category, based on NOVA and World Health Organisation Regional Office for Europe Nutrient Profile model (WHO-model) classifications. ND = No data; not enough information to classify as (non-)permitted without nutritional data. *Assessment of the commitments and performance of the European food industry to improve population nutrition, Europe, 2020.*

| **Euromonitor**  **food groups** | **NOVA** | **WHO-model** | **Euromonitor**  **food groups** | **NOVA** | **WHO-model** |
| --- | --- | --- | --- | --- | --- |
| **Edible Oils** | Non-ultra-processed | ND | **Multi-Pack Water Ice Cream** | Ultra-processed | Non-permitted |
| **Shelf Stable Ready Meals** | Ultra-processed | ND | **Nuts, Seeds and Trail Mixes** | Non-ultra-processed | ND |
| **Chilled Lunch Kits** | Ultra-processed | ND | **Potato Chips** | Ultra-processed | ND |
| **Chilled Pizza** | Ultra-processed | ND | **Tortilla Chips** | Ultra-processed | ND |
| **Chilled Ready Meals** | Ultra-processed | ND | **Puffed Snacks** | Ultra-processed | ND |
| **Dinner Mixes** | Ultra-processed | ND | **Rice Snacks** | Ultra-processed | ND |
| **Dried Ready Meals** | Ultra-processed | ND | **Vegetable, Pulse and Bread Chips** | Ultra-processed | ND |
| **Frozen Pizza** | Ultra-processed | ND | **Savoury Biscuits** | Ultra-processed | ND |
| **Frozen Ready Meals** | Ultra-processed | ND | **Popcorn** | Ultra-processed | ND |
| **Prepared Salads** | Non-ultra-processed | ND | **Pretzels** | Ultra-processed | ND |
| **Gravy Cubes and Powders** | Ultra-processed | ND | **Other Savoury Snacks** | Ultra-processed | ND |
| **Liquid Stocks and Fonds** | Ultra-processed | ND | **Dried Fruit** | Non-ultra-processed | ND |
| **Stock Cubes and Powders** | Ultra-processed | ND | **Processed Fruit Snacks** | Ultra-processed | ND |
| **Dry Sauces** | Ultra-processed | ND | **Cereal Bars** | Ultra-processed | Non-permitted |
| **Herbs and Spices** | Non-ultra-processed | ND | **Energy Bars** | Ultra-processed | Non-permitted |
| **Monosodium Glutamate** | Ultra-processed | ND | **Fruit and Nut Bars** | Non-ultra-processed | Non-permitted |
| **Pasta Sauces** | Ultra-processed | ND | **Other Snack Bars** | Ultra-processed | Non-permitted |
| **Cooking Sauces** | Ultra-processed | ND | **Chocolate Coated Biscuits** | Ultra-processed | Non-permitted |
| **Dips** | Ultra-processed | ND | **Cookies** | Ultra-processed | Non-permitted |
| **Pickled Products** | Ultra-processed | ND | **Filled Biscuits** | Ultra-processed | Non-permitted |
| **Barbecue Sauces** | Ultra-processed | ND | **Plain Biscuits** | Ultra-processed | Non-permitted |
| **Fish Sauces** | Ultra-processed | ND | **Wafers** | Ultra-processed | Non-permitted |
| **Ketchup** | Ultra-processed | ND | **Packaged Flat Bread** | Non-ultra-processed | ND |
| **Mayonnaise** | Ultra-processed | ND | **Unpackaged Flat Bread** | Non-ultra-processed | ND |
| **Mustard** | Ultra-processed | ND | **Packaged Leavened Bread** | Ultra-processed | ND |
| **Oyster Sauces** | Ultra-processed | ND | **Unpackaged Leavened Bread** | Non-ultra-processed | ND |
| **Salad Dressings** | Ultra-processed | ND | **Packaged Cakes** | Ultra-processed | Non-permitted |
| **Soy Sauces** | Ultra-processed | ND | **Unpackaged Cakes** | Non-ultra-processed | Non-permitted |
| **Chili Sauces** | Ultra-processed | ND | **Dessert Mixes** | Ultra-processed | Non-permitted |
| **Other Table Sauces** | Ultra-processed | ND | **Frozen Baked Goods** | Ultra-processed | Non-permitted |
| **Tomato Pastes and Purées** | Ultra-processed | ND | **Packaged Pastries** | Ultra-processed | Non-permitted |
| **Yeast-based Spreads** | Ultra-processed | ND | **Unpackaged Pastries** | Non-ultra-processed | Non-permitted |
| **Other Sauces, Dressings and Condiments** | Ultra-processed | ND | **Hot Cereals** | Non-ultra-processed | ND |
| **Shelf Stable Soup** | Ultra-processed | ND | **Children's Breakfast Cereals** | Ultra-processed | Non-permitted |
| **Chilled Soup** | Ultra-processed | ND | **Flakes** | Ultra-processed | ND |
| **Dehydrated Soup** | Ultra-processed | ND | **Muesli and Granola** | Ultra-processed | ND |
| **Frozen Soup** | Ultra-processed | ND | **Other RTE Cereals** | Ultra-processed | ND |
| **Instant Soup** | Ultra-processed | ND | **Shelf Stable Beans** | Non-ultra-processed | ND |
| **Honey** | Non-ultra-processed | Non-permitted | **Shelf Stable Fruit** | Non-ultra-processed | ND |
| **Chocolate Spreads** | Ultra-processed | Non-permitted | **Shelf Stable Tomatoes** | Non-ultra-processed | ND |
| **Jams and Preserves** | Ultra-processed | Not-permitted | **Shelf Stable Vegetables** | Non-ultra-processed | ND |
| **Nut and Seed Based Spreads** | Ultra-processed | Non-permitted | **Frozen Fruit** | Non-ultra-processed | ND |
| **Butter** | Non-ultra-processed | ND | **Frozen Processed Potatoes** | Ultra-processed | ND |
| **Cooking Fats** | Non-ultra-processed | ND | **Frozen Processed Vegetables** | Non-ultra-processed | ND |
| **Margarine and Spreads** | Ultra-processed | ND | **Shelf Stable Processed Red Meat** | Ultra-processed | ND |
| **Spreadable Processed Cheese** | Ultra-processed | ND | **Shelf Stable Processed Poultry** | Ultra-processed | ND |
| **Other Processed Cheese** | Ultra-processed | ND | **Chilled Processed Red Meat** | Non-ultra-processed | ND |
| **Packaged Hard Cheese** | Non-ultra-processed | ND | **Chilled Processed Poultry** | Non-ultra-processed | ND |
| **Unpackaged Hard Cheese** | Non-ultra-processed | ND | **Frozen Processed Red Meat** | Non-ultra-processed | ND |
| **Soft Cheese** | Non-ultra-processed | ND | **Frozen Processed Poultry** | Non-ultra-processed | ND |
| **Dairy Only Flavoured Milk Drinks** | Ultra-processed | Non-permitted | **Shelf Stable Seafood** | Ultra-processed | ND |
| **Flavoured Milk Drinks with Fruit Juice** | Ultra-processed | Non-permitted | **Chilled Processed Seafood** | Non-ultra-processed | ND |
| **Fresh Milk** | Non-ultra-processed | ND | **Frozen Processed Seafood** | Non-ultra-processed | ND |
| **Shelf Stable Milk** | Non-ultra-processed | ND | **Chilled Meat Substitutes** | Ultra-processed | ND |
| **Goat Milk** | Non-ultra-processed | ND | **Frozen Meat Substitutes** | Ultra-processed | ND |
| **Powder Milk** | Non-ultra-processed | ND | **Shelf Stable Meat Substitutes** | Ultra-processed | ND |
| **Soy Drinks** | Ultra-processed | ND | **Chilled Noodles** | Non-ultra-processed | ND |
| **Other Milk Alternatives** | Ultra-processed | ND | **Instant Noodle Cups** | Ultra-processed | ND |
| **Sour Milk Products** | Non-ultra-processed | ND | **Instant Noodle Pouches** | Ultra-processed | ND |
| **Drinking Yoghurt** | Ultra-processed | ND | **Plain Noodles** | Non-ultra-processed | ND |
| **Flavoured Yoghurt** | Ultra-processed | ND | **Chilled Pasta** | Non-ultra-processed | ND |
| **Plain Yoghurt** | Non-ultra-processed | ND | **Dried Pasta** | Non-ultra-processed | ND |
| **Chilled Dairy Desserts** | Ultra-processed | Non-permitted | **Rice** | Non-ultra-processed | ND |
| **Shelf Stable Dairy Desserts** | Ultra-processed | Non-permitted | **Carbonated Natural Mineral Bottled Water** | Non-ultra-processed | ND |
| **Chilled Snacks** | Ultra-processed | ND | **Carbonated Spring Bottled Water** | Non-ultra-processed | ND |
| **Coffee Whiteners** | Ultra-processed | ND | **Carbonated Purified Bottled Water** | Non-ultra-processed | ND |
| **Flavoured Condensed Milk** | Ultra-processed | ND | **Flavoured Bottled Water** | Ultra-processed | Non-permitted |
| **Plain Condensed Milk** | Non-ultra-processed | ND | **Functional Bottled Water** | Non-ultra-processed | ND |
| **Cream** | Non-ultra-processed | ND | **Still Natural Mineral Bottled Water** | Non-ultra-processed | ND |
| **Flavoured Fromage Frais and Quark** | Non-ultra-processed | ND | **Still Spring Bottled Water** | Non-ultra-processed | ND |
| **Plain Fromage Frais and Quark** | Non-ultra-processed | ND | **Still Purified Bottled Water** | Non-ultra-processed | ND |
| **Savoury Fromage Frais and Quark** | Non-ultra-processed | ND | **Low Calorie Cola Carbonates** | Ultra-processed | Non-permitted |
| **Chocolate Pouches and Bags** | Ultra-processed | Non-permitted | **Regular Cola Carbonates** | Ultra-processed | Non-permitted |
| **Boxed Assortments** | Ultra-processed | Non-permitted | **Lemonade/Lime** | Ultra-processed | Non-permitted |
| **Chocolate with Toys** | Ultra-processed | Non-permitted | **Ginger Ale** | Ultra-processed | Non-permitted |
| **Countlines** | Ultra-processed | Non-permitted | **Tonic Water/Other Bitters** | Ultra-processed | Non-permitted |
| **Seasonal Chocolate** | Ultra-processed | Non-permitted | **Orange Carbonates** | Ultra-processed | Non-permitted |
| **Tablets** | Ultra-processed | Non-permitted | **Other Non-Cola Carbonates** | Ultra-processed | Non-permitted |
| **Other Chocolate Confectionery** | Ultra-processed | Non-permitted | **Liquid Concentrates** | Ultra-processed | Non-permitted |
| **Bubble Gum** | Ultra-processed | Non-permitted | **Powder Concentrates** | Ultra-processed | Non-permitted |
| **Chewing Gum** | Ultra-processed | Non-permitted | **Not from Concentrate 100% Juice** | Non-ultra-processed | Non-permitted |
| **Boiled Sweets** | Ultra-processed | Non-permitted | **Reconstituted 100% Juice** | Ultra-processed | Non-permitted |
| **Liquorice** | Ultra-processed | Non-permitted | **Juice Drinks (up to 24% Juice)** | Ultra-processed | Non-permitted |
| **Lollipops** | Ultra-processed | Non-permitted | **Nectars** | Ultra-processed | Non-permitted |
| **Medicated Confectionery** | Ultra-processed | Non-permitted | **Coconut and Other Plant Waters** | Non-ultra-processed | ND |
| **Power Mints** | Ultra-processed | Non-permitted | **RTD Coffee** | Ultra-processed | Non-permitted |
| **Standard Mints** | Ultra-processed | Non-permitted | **Carbonated RTD Tea** | Ultra-processed | Non-permitted |
| **Pastilles, Gums, Jellies and Chews** | Ultra-processed | Non-permitted | **Still RTD Tea** | Ultra-processed | Non-permitted |
| **Toffees, Caramels and Nougat** | Ultra-processed | Non-permitted | **Energy Drinks** | Ultra-processed | Non-permitted |
| **Other Sugar Confectionery** | Ultra-processed | Non-permitted | **Sports Drinks** | Ultra-processed | Non-permitted |
| **Frozen Desserts** | Ultra-processed | Non-permitted | **Asian Speciality Drinks** | Ultra-processed | Non-permitted |
| **Frozen Yoghurt** | Ultra-processed | Non-permitted |  |  |  |
| **Single Portion Dairy Ice Cream** | Ultra-processed | Non-permitted |  |  |  |
| **Single Portion Water Ice Cream** | Ultra-processed | Non-permitted |  |  |  |
| **Unpackaged Ice Cream** | Ultra-processed | Non-permitted |  |  |  |
| **Bulk Dairy Ice Cream** | Ultra-processed | Non-permitted |  |  |  |
| **Ice Cream Desserts** | Ultra-processed | Non-permitted |  |  |  |
| **Multi-Pack Dairy Ice Cream** | Ultra-processed | Non-permitted |  |  |  |
| **Bulk Water Ice Cream** | Ultra-processed | Non-permitted |  |  |  |

Supplementary file 4: Examples of how publicly available commitments were collected and scored according to the Business Impact Assessment on Obesity and Population Level Nutrition (BIA-Obesity) tool, Europe, 2020. *Assessment of the commitments and performance of the European food industry to improve population nutrition, Europe, 2020.*

| **Domain** | **Indicator** | **Policy content** | **Scoring criteria** | **score** |
| --- | --- | --- | --- | --- |
| Corporate nutrition strategy | *Does the company have an overarching commitment to improving population nutrition and health articulated in strategic documents (e.g., corporate strategy document, corporate responsibility reports)?* | *“Our mission is to bring health through food to as many people as possible. We have created a unique portfolio of healthy products to complete this mission, and we strive to continuously optimize their nutritional profile.” - Danone* | *10: Yes, a specific commitment to improving population nutrition and health, at the European level or at the global level with reference to the European market or multiple European countries, publicly available in strategic documents*  *7.5: Yes, a specific global commitment to improving population nutrition and health, publicly available in strategic documents*  *5: Yes, a European- or global- level commitment, but not publicly-available, OR general reference to nutrition and health as part of general corporate strategy*  *0: No clear commitments to improving population nutrition and health* | *7.5* |
| Product formulation | *Has the company set a target/targets or provided detailed evidence of having taken significant action to reduce/reach lower levels of added sugars, and is it applicable to Europe?* | *“1. By 2020, we will remove 25% of sugar from our ready-to-drink tea products, as set out in our position statement on sugar. To meet this stretching target, we developed more drinks that meet our Highest Nutritional Standards (HNS) of 5g or less sugar per 100ml. And by 2018, we had removed 20% of sugar across all our sweetened tea-based beverages (against a 2010 baseline).*  *2. We focus on beverages and ice cream because that is where we can have the biggest impact on sugar reduction and therefore public health.” - Unilever* | *10: Set SMART targets or provided detailed evidence of having taken significant action in all key categories/subcategories, published*  *5: Targets (not necessarily SMART) set or taken significant action in some key products/sub-categories / not published*  *2.5: General or vague commitment to reducing use of added sugars in products, published or disclosed to INFORMAS team*  *0: No target / no information* | *5* |
| Nutrition labelling | *Does the company have a published commitment to rolling out a government endorsed FOP labelling system (e.g. NutriScore, Traffic light)?* | *“We aim to implement Nutri-Score at scale, starting in countries that already support the scheme, such as France, Belgium, Switzerland and Germany. Constructive engagement will continue in other countries to ensure the best possible outcome for all Europeans.” - Nestlé* | *10: Yes, with implementation plan across all product categories (published or unpublished)*  *7.5: Yes, with implementation plan across a selection of product categories (published or unpublished)*  *5: Yes, but with no specific implementation plan (published or unpublished)*  *0: No* | *10* |
| Product and brand promotion | *Does the company have an explicit policy to reduce the exposure of children to unhealthy food marketing on broadcast media (TV, radio)?*  *(Note: check if the company supports the EU Pledge. If yes and no other comments, then EU pledge is scored)* | *“The Intersnack Group is a member of the European Snacks Association (ESA) and a signatory of the EU Pledge, a voluntary initiative by leading food and beverage companies to change food and beverage advertising to children under the age of twelve in the European Union.” - Intersnack Knabber-Gebäck* | *10: Yes, European policy or policy that refers to multiple European countries and noted on company website / annual reports*  *7.5: Yes, global policy and noted on company website / annual reports*  *5: Yes, European policy or policy that refers to multiple European countries, but not noted on company website / annual reports OR noted on industry association website*  *2.5: Yes, global policy but not noted on company website / annual reports*  *0: No policy/ no information available to the research team* | *10* |
| Product accessibility | *Does the company publish its policy position (in relation to government policy) on fiscal policies to make healthier foods relatively cheaper and unhealthy foods relatively more expensive?* | *“Obesity and NCDs are extremely complex problems and the right answers aren’t always the simple ones. Experience from around the world shows no evidence that a tax on soft drinks helps to reduce obesity. We’re determined to help create a healthy food environment in Europe and we are committed to supporting and accelerating what works, which is why reducing sugar from our drinks is such a top priority. We’ve already seen consumer behavior changing, but we know there is much more work to be done.” – Coca-Cola* | *10: Yes, on own website*  *5: Yes, on industry association website*  *0: Not publicly available* | *10* |
| Relationships with other organisations | *Does the company publish details of the nutrition education / healthy diet oriented programs it funds or supports?* | *“1. ‘Partnership for Health’ is a programme designed by four partners: the Institute of Mother and Child and the following companies: Danone, Biedronka and Lubella. “Partnership for Health” is a unique initiative on the Polish market. The three commercial companies and the Institute started a joint initiative in order to tackle the problem of an unbalanced diet of Polish children and its dramatic effects on health and society.*  *2. This educational program is addressed to students in the sixth-eighth grades of primary school and third grade of junior high school and their teachers. In the school year 2018/19, as many as 160,000 pupils from primary and junior high schools from all over Poland took part in it! They gained not only extensive knowledge about healthy lifestyle, nutrition principles, lack of food and cooking, but also participated in special competitions in which attractive prizes were available!﻿﻿” - Maspex Wadowice* | *10: Yes, information on European activity or activity in multiple European countries is publicly available (website or document) in a consolidated and cumulative form OR active declaration/policy stating no activity in this area (either publicly available or disclosed to INFORMAS team)*  *5: Yes, information is available, but is not consolidated and easy to locate OR information is available at the global level only OR comprehensive information about their activities in the area provided to the project team*  *0: No information available / provided* | *5* |

Supplementary file 5: Weighting per ‘Business Impact Assessment on Obesity and Population Nutrition’ (BIA-Obesity) domain and food industry. *Assessment of the commitments and performance of the European food industry to improve population nutrition, Europe, 2020.*

| **BIA-Obesity domains** | **Packaged food and Soft drinks** | **Chain restaurants** | **Supermarkets** |
| --- | --- | --- | --- |
| Corporate nutrition strategy | 10 | 10 | 10 |
| Product formulation | 30 | 25 | 25 |
| Nutrition labelling | 20 | 15 | 15 |
| Product and brand promotion | 30 | 25 | 25 |
| Product accessibility | 5 | 20 | 20 |
| Relationships with other organisations | 5 | 5 | 5 |
| **TOTAL** | 100 | 100 | 100 |
