# Supplementary material for: Assessment of the Commitments and Performance of the European Food Industry to Improve Population Nutrition
Source: Int J Public Health. 2022 Jun 1;67:1604116. doi: 10.3389/ijph.2022.1604116 (PMC9198223; doi:10.3389/ijph.2022.1604116)
Supplement: Supplementary file 2 [file DataSheet1.docx]

**Supplementary file 1: The ‘Business Impact Assessment on Obesity and Population Level Nutrition’ (BIA-Obesity) tool and process to assess food company policies and commitments related to obesity prevention and population nutrition – Adaptation for Europe. Assessment of the commitments and performance of the European food industry to improve population nutrition, Europe, 2020.**

Table of Contents

[**Table S1 – Phase 1: Indicators and scoring criteria for packaged food and non-alcoholic beverage manufacturers** 2](#_Toc67659214)

[**Table S2 – Phase 1: Indicators and scoring criteria for supermarkets** 21](#_Toc67659215)

[**Table S3 – Phase 1: Indicators and scoring criteria for chain restaurants** 43](#_Toc67659216)

## **Table S1 – Phase 1: Indicators and scoring criteria for packaged food and non-alcoholic beverage manufacturers**

| **Indicator ID** | **Domain and indicator** | **Scoring** |
| --- | --- | --- |
| **M-STRAT** | **Corporate nutrition strategy** | **Maximum total points = 30** |
| M-STRAT1 | Does the company have an overarching commitment to improving population nutrition and health articulated in strategic documents (e.g., corporate strategy document, corporate responsibility reports)? | 10: Yes, a specific commitment to improving population nutrition and health, at the European level or at the global level with reference to the European market or multiple European countries, publicly available in strategic documents  7.5: Yes, a specific global commitment to improving population nutrition and health, publicly available in strategic documents  5: Yes, a European- or global- level commitment, but not publicly-available, OR general reference to nutrition and health as part of general corporate strategy  0: No clear commitments to improving population nutrition and health |
| M-STRAT2 | Does the company’s commitment to improving population nutrition and health (where it exists) include specific objectives and targets for obesity and NCDs (including for improving population nutrition)? | (Can be multiple, max of 10 points)  2: Contains specific, measurable, achievable, relevant and time bound (SMART) objectives and targets  2: Recognition or reference to relevant priorities set out in the WHO Global Action Plan for the Prevention and Control of NCDs 2013 - 2020, the WHO European Food and Nutrition Action Plan 2015 – 2020, the Sustainable Development Goals, or the WHO Report on Ending Childhood Obesity  2: Recognition or reference to relevant priorities in European policy documents relating to population nutrition and obesity/NCD prevention (e.g. A Strategy for Europe on Nutrition, Overweight and Obesity related health issues; EU Action Plan on Childhood Obesity 2014-2020; Horizon 2020; Fruit and vegetable regime)  2: Comprehensive in nature (e.g., includes three or more domains in this document, such as formulation, marketing and labelling)  2: Key Performance Indicators (KPIs) (and/or remuneration) of management linked to nutrition strategy/policy/targets |
| M-STRAT3 | Does the company regularly publish details of its approach to population nutrition and health related to obesity and NCDs? | 10: Regular, publicly available reports including reporting against objectives and targets, a clear outlook of future plans and challenges, external verification / review, and that specifically refers to Europe or multiple European countries  7.5: Regular, publicly available global reports with no specific reference to Europe or European countries  5: Regular reports including some of the relevant information  2.5: Irregular reporting  0: None published |
|  | | |
| **M-FORM** | **Product formulation** | **Maximum total points = 95** |
| M-FORM1 | Does the company publish a comprehensive set of commitments or objectives related to new product development and reformulating its existing products with respect to reducing the nutrients of concern and energy (salt/sodium, saturated fats, trans fats, added sugar and kilojoules/portion size)? | 10: Yes, specific European commitments/objectives or specific global commitments/objectives that include specific reference to multiple European countries, publicly available  7.5: Yes, specific global commitments/objectives that are publicly available  5: Has specific European commitments/objectives or specific global commitments/objectives with reference to multiple European countries, but not publicly available  2.5: Has European or global-level commitments/objectives in this area that are available publicly, but these commitments/objectives are vague and non-specific OR has global commitments/objectives but not publicly available  0: No commitment/ no policy information available to the research team |
| M-FORM2 | Is the company a signatory to European and/or global industry initiatives on product reformulation or do they commit to voluntary programs on product reformulation?  *(e.g. IFBA commitments on reformulation)* | 5: Yes, and noted on company website / annual reports  2.5 Yes, but not noted on company website / annual reports (e.g. government/ NGO/ industry organisation’s website or disclosed directly to INFORMAS)  0: No / no information |
|  | **Salt/sodium** (only assess where relevant to a company’s product portfolio and if not mandatory according to European government policy)  REMARK: In some EU countries there may be policies in place | |
| M-FORM3.1 | Has the company set a target/targets or provided detailed evidence of having taken significant action to reduce/reach lower levels of salt/sodium in products, and is it applicable to Europe? | 10: Set SMART targets or provided detailed evidence of having taken significant action in all key categories/subcategories, published  5: Targets (not necessarily SMART) set or significant action taken in some key products/sub-categories/not published  2.5: General or vague commitment to reducing levels of salt/sodium in products, published or disclosed to INFORMAS team  0: No target / no information |
| M-FORM3.2 | When is the baseline year and target year?  What are the targets? | [Information only, not to be scored] |
|  | **Trans and saturated fats** (only assess where relevant to a company’s product portfolio and if not mandatory according to European government policy, not applicable to beverage industry)  REMARK: In some EU countries there may be policies in place, e.g. Denmark | |
| M-FORM4.1 | Has the company set a target/targets or provided detailed evidence of having taken significant action to reduce artificial trans fat added to products during the manufacturing process, and is it applicable to Europe?  *!Keep in mind possible upcoming EU regulation on max content of trans fats in foods (open for consultation)* | 10: Set a target or provided detailed evidence of having taken significant action to eliminate trans fat in all relevant categories/subcategories, published  5: Set a target or taken significant action to eliminate/reduce in some relevant products/sub-categories/not published  2.5: General or vague commitment to reducing/eliminating use of trans fats in products, published or disclosed to INFORMAS team  0: No target / no information |
| M-FORM4.2 | When is the baseline year and target year?  What are the targets? | [Information only, not to be scored] |
| M-FORM5.1 | Has the company set a target/targets or provided detailed evidence of having taken significant action to reduce/reach lower levels of saturated fats, and is it applicable to Europe? | 10: Set SMART targets or provided detailed evidence of having taken significant action in all key categories/subcategories, published  5: Targets (not necessarily SMART) set or taken significant action in some key products/sub-categories/not published  2.5: General or vague commitment to reducing use of saturated fats in products, published or disclosed to INFORMAS team  0: No target / no information |
| M-FORM5.2 | When is the baseline year and target year?  What are the targets? | [Information only, not to be scored] |
|  | **Added sugars** (only assess where relevant to a company’s product portfolio and if not mandatory according to government policy) | |
| M-FORM6.1 | Has the company set a target/targets or provided detailed evidence of having taken significant action to reduce/reach lower levels of added sugars, and is it applicable to Europe? | 10: Set SMART targets or provided detailed evidence of having taken significant action in all key categories/subcategories, published  5: Targets (not necessarily SMART) set or taken significant action in some key products/sub-categories / not published  2.5: General or vague commitment to reducing use of added sugars in products, published or disclosed to INFORMAS team  0: No target / no information |
| M-FORM6.2 | When is the baseline year and target year?  What are the targets? | [Information only, not to be scored] |
|  | **Portion size (energy content)** (only assess where relevant to a company’s product portfolio and if not mandatory according to government policy) | |
| M-FORM7.1 | Does the company have a target/targets or provided detailed evidence of having taken significant action to reduce the portion size / energy content of single serve snacks, and is it applicable to Europe? | 10: Set SMART targets or provided detailed evidence of having taken significant action in all key categories/subcategories, published  5: Targets (not necessarily SMART) set or taken significant action in some key products/sub-categories / not published  2.5: General or vague commitment to reducing portion size / energy content in products, published or disclosed to INFORMAS team  0: No target / no information |
| M-FORM7.2 | When is the baseline year and target year?  What are the targets? | [Information only, not to be scored] |
|  | **Classification system** |  |
| M-FORM8.1 | What system / criteria (e.g., product classification system or nutrient profiling system) does the company use to classify the healthiness of products for the purposes of **product development / reformulation**? | 10: Uses government guidelines/government endorsed classification system (where available e.g. WHO Europe nutrient profile model)  7.5**: Publicly** available system, developed in consultation with experts and in line with government guidelines, published in peer reviewed literature  5: **Publicly** available system, developed in consultation with experts and in line with government guidelines (not published in peer reviewed literature)  2.5: **Publicly** available system with no details of development/alignment with government guidelines OR **not** **publicly** available but developed in consultation with experts and aligned with government guidelines  0: No information / poor alignment / does not have a system |
| M-FORM8.2 | If a proprietary product classification system has been developed, which products, nutrients and food characteristics are covered, and what are the details? | [Information only, not to be scored] |
|  | **Policy position on reformulation** | |
| M-FORM9.1 | Does the company publish its policy position (in relation to government policy) on **product reformulation?** | 10: Yes, on own website  5: Yes, on industry association website  2.5: Policy position made available to INFORMAS team  0: Not publicly available |
| M-FORM9.2 | Does the company’s policy position support WHO’s position on **product reformulation** in relation to nutrients of concern, as articulated in the Global Action Plan for the Prevention and Control of NCDs 2013-2020 or the WHO European Food and Nutrition Action Plan 2015 – 2020? | 10: Support for government-led efforts to reformulate the food supply in relation to several nutrients of concern  5: Support for government-led efforts to reformulate the food supply in relation to only one nutrient of concern  0: No details available  -5: Somewhat opposed (e.g., opposes some aspects of implementation of government-led efforts to reformulate the food supply)  -10: Opposed to government-led efforts to reformulate the food supply in relation to nutrients of concern |
| **M-LABEL** | **Nutrition labelling** | **Maximum total points = 90** |
|  | **General nutrition labelling information** | |
| M-LABEL1 | Does the company commit to provide on-pack information on *trans-*fat content? | 2.5: Yes, on all relevant products  1: Yes, on some products  0: No commitment / no information available to the research team  N/A: if commitment to eliminate use of all industrially produced trans-fat across portfolio |
| M-LABEL2 | Does the company commit to provide on-pack information on added sugar content? | 2.5: Yes, on all relevant products  1: Yes, on some products  0: No commitment / no information available to the research team |
| M-LABEL3 | Does the company provide nutrition information online for food products within its portfolio? | 10: Yes, comprehensive nutrition information (calories, sodium, saturated fat, total fat, sugar) for most (>80%) products, including on a per 100g/100ml basis  7.5: Yes, comprehensive nutrition information (calories, sodium, saturated fat, total fat, sugar) for most (>80%) products, or comprehensive nutrition information for all products per serving only  5: Comprehensive nutrition information for some (>50%) products  2.5: Limited nutrition information (i.e. does not include calories, sodium, saturated fat, total fat or sugar) for some (>50%) products  0: <50% of products or no information |
| M-LABEL4 | Does the company have a policy to provide information on food composition to the EU commission , on request?  (if applicable, e.g., information has been requested by government) | 5: Yes, all products (published or not published)  2.5: Yes, some products  0: No policy / no information available to the research team |
|  | **Front of pack (FOP) nutrition labelling** | |
| M-LABEL5.1 | **IF A VOLUNTARY GOVERNMENT-ENDORSED FOP NUTRITION LABELLING SCHEME EXISTS:**  Does the company have a published commitment to rolling out a government endorsed FOP labelling system (e.g. NutriSCore, Traffic light)? | 10: Yes, with implementation plan across all product categories (published or unpublished)  7.5: Yes, with implementation plan across a selection of product categories (published or unpublished)  5: Yes, but with no specific implementation plan (published or unpublished)  0: No |
| M-LABEL5.2 | If the company **does not commit to full implementation of a government endorsed FOP labelling system**, what FOP labelling system does the company use?  Remark: Own FOP labelling systems do hamper the development and implementation of government endorsed FOP labelling systems | 10: Interpretive information (such as, stars, traffic lights, warning labels, etc.), applied across all product categories  7.5: Symbols or logos that indicate healthy products, applied across all product categories  5: Numeric information with % of recommended daily intake, applied across all product categories  2.5: Numeric information on levels of key nutrients, not showing % of recommended daily intake, applied across all product categories  0: No FOP labelling used  **DIVIDE POINTS IN HALF IF ONLY USED FOR SOME PRODUCTS / CATEGORIES**  **N/A if full implementation of government endorsed system** |
| M-LABEL6.1 | Does the company publish its policy position (in relation to government policy) on front of pack nutrition labelling? | 10: Yes, on own website  5: Yes, on industry association website  2.5: Policy position made available to INFORMAS team  0: Not publicly available |
| M-LABEL6.2 | Does the company’s policy position (in relation to government policy) support WHO’s position on front of pack nutrition labelling, as articulated in the WHO Report of the Commission on Ending Childhood Obesity (Recommendation 1.7) or the WHO European Food and Nutrition Action plan 2015 - 2020?  *(Statement on Recommendation 1.7: Implement interpretive front-of-pack labelling, supported by public education of both adults and children for nutrition literacy)*  *(EU Action plan: Increase consumer-friendly labelling by establishing easy-to-understand or interpretative front - of package labels that help consumers to identify healthier options)* | 10: Strong support (with a focus on “interpretive” to mean readily understandable and providing easy comparison of products e.g. using traffic light colours or stars to compare products for those with low nutritional literacy; broadly implemented)  5: Weak support (e.g., supports scheme that only provides limited interpretive information, such as % Daily Intake Guide for energy content only)  0: No details available  -5: Somewhat opposed (e.g., opposes some aspects of implementation of an interpretive scheme)  -10: Strongly opposed (e.g., opposes interpretive labelling) |
|  | **Health and nutrition claims** | |
| M-LABEL7 | Does the company state that it will place a health claim on a product (or use a health claim as part of product advertising) only when the product is 'healthy'? | 10: Yes, commitment is published  5: Yes, commitment is not published  0: No commitment/ no information available to the research team |
| M-LABEL8 | Does the company state that it will place a nutrition claim on a product (or use a nutrition claim as part of product advertising) only when the product is 'healthy'? | 10: Yes, commitment is published  5: Yes, commitment is not published  0: No commitment/ no information available to the research team |
| M-LABEL9.1 | What system / criteria (e.g., product classification system or nutrient profiling system) does the company use to classify the healthiness of products for the purposes of **health and/or nutrition claims?** | 10: Adopted an official classification system (developed by WHO, PAHO, national government, etc.)  5: Developed own system that has been validated and shows strong alignment with official classification systems / dietary guidelines, published in peer-reviewed literature  2.5: Developed own system that has been validated and shows alignment with official classification systems / dietary guidelines, not published in peer-reviewed literature  0: No information / poor alignment / does not have a system |
| M-LABEL9.2 | If a proprietary product classification system has been developed, which products, nutrients and food characteristics are covered, and what are the details? | [Information only, not to be scored] |
|  | | |
| **M-PROMO** | **Product and brand promotion** | **Maximum total points = 120** |
|  | **Broadcast media** |  |
| M-PROMO1.1 | Does the company have an explicit policy to reduce the exposure of children to unhealthy food marketing on broadcast media (TV, radio)?  *(Note: check if the company supports the EU Pledge. If yes and no other comments, then EU pledge is scored)* | 10: Yes, European policy or policy that refers to multiple European countries and noted on company website / annual reports  7.5: Yes, global policy and noted on company website / annual reports  5: Yes, European policy or policy that refers to multiple European countries, but not noted on company website / annual reports OR noted on industry association website  2.5: Yes, global policy but not noted on company website / annual reports  0: No policy/ no information available to the research team |
| M-PROMO1.2 | To what age group(s) does the broadcast marketing policy apply?  *(Note: check if the company supports the EU Pledge. If yes and no other comments, then EU pledge is scored)* | 10: 18 years and under  8: 16 years and under  6: 14 years and under  4: 12 years and under  2: Under 10 years  0: No policy / no information |
| M-PROMO1.3 | How is the ‘target audience’ or ‘audience exposed’ defined?  *(Note: check if the company supports the EU Pledge. If yes and no other comments, then EU pledge is scored)* | 10: Time-based restrictions, based on children’s peak viewing times (e.g., no advertising before 9:00pm)  5: Based on a percentage of the children’s audience that are likely to be viewing (e.g. if >10% of total children are watching)  2.5: Based on a percentage of the audience that are likely to be children (e.g., if >10% of audience are children)  1: Children’s programmes only  0: No explicit threshold / definition |
|  | **Non-broadcast media** | |
| M-PROMO2.1 | Does the company have an explicit policy to reduce the exposure of children to unhealthy food marketing on non-broadcast media (including websites, DVDs/games, social media, print media, product placement, outdoor marketing (school zones excluded - under PROMO4), in store marketing / point of sales marketing)?  *(Note: check if the company supports the EU Pledge. If yes and no other comments, then EU pledge is scored)* | 10: Yes, European policy or policy that refers to multiple European countries and noted on company website / annual reports  7.5: Yes, global policy and noted on company website / annual reports  5: Yes, European policy or policy that refers to multiple European countries, but not noted on company website / annual reports OR noted on industry association website  2.5: Yes, global policy but not noted on company website / annual reports  0: No policy/ no information available to the research team |
| M-PROMO2.2 | To what age group(s) does the non-broadcast marketing policy apply?  *(Note: check if the company supports the EU Pledge. If yes and no other comments, then EU pledge is scored)* | 10: 18 years and under  8: 16 years and under  6: 14 years and under  4: 12 years and under  2: Under 10 years  0: No policy / no information |
| M-PROMO3 | Does the company commit not to sponsor children’s sporting, cultural or other activities using unhealthy brands (foods or company brands)?  *(Note: check if the company supports the EU Pledge. If yes and no other comments, then EU pledge is scored)* | 10: Yes, comprehensive commitment including products and brands  5: Yes, comprehensive commitment including products only (brands still permitted)  2.5: Some commitments in the area, including some events or some forms of sponsorship  0: No commitment / no information available to the research team |
| M-PROMO4 | Does the company commit not to use marketing in settings where children gather using unhealthy brands (foods or company brands)?  *(Note: check if the company supports the EU Pledge. If yes and no other comments, then EU pledge is scored)* | (Can be multiple)  2: Commits IN early childcare settings and primary schools (children up to age 11)  2: Commits NEAR (e.g. within 500m) of early childcare settings and primary schools (children up to age 11)  2: Commits IN secondary schools (children between age 12 and 18)  2: Commits NEAR (e.g., within 500m) of secondary schools (children between age 12 and 18)  2: Commits in other places where children gather (family and child clinics, paediatric services or other health facilities, sporting or recreation centres, or sporting or cultural events held at those premises) |
|  | **General policies regarding promotion to children** | |
| M-PROMO5.1 | Does the company pledge not to use celebrities in marketing of products to children other than those that meet the company’s healthy standard?  *(Note: check if the company supports the EU Pledge. If yes and no other comments, then EU pledge is scored)* | 5: All forms of marketing  2.5: Some forms of marketing (e.g., excludes packaging) or applies only to those celebrities that appeal primarily to children  0: No policy / no information available to the research team |
| M-PROMO5.2 | Does the company pledge not to use fantasy and animation characters with a strong appeal to children in marketing of products other than those that meet the company’s healthy standard?  *(Note: check if the company supports the EU Pledge. If yes and no other comments, then EU pledge is scored)* | 5: All forms of marketing (includes no use of characters with strong appeal to children across **all** forms of marketing)  2.5: Some forms of marketing (includes no use of characters with strong appeal to children across **some** forms of marketing)  0: No policy / no information available to the research team |
| M-PROMO5.3 | Does the company commit to not use premium offers (e.g., promotional toys, games, vouchers and competitions) in marketing of products other than those that meet the company’s healthy standard?  *(Note: check if the company supports the EU Pledge. If yes and no other comments, then EU pledge is scored)* | 5: All forms of marketing  2.5: Some forms of marketing (e.g., excludes packaging)  0: No commitment / no information available to the research team |
| M-PROMO6 | Does the company audit/monitor its compliance with its policy on marketing to children at the?  *(Note: check if the company supports the EU Pledge. If yes and no other comments, then EU pledge is scored)* | 5: Yes, the policy is audited by an independently appointed third party  2.5: Yes, the policy independently audited  1: No, the policy is not independently audited  0: No auditing is conducted |
|  | **Classification system** |  |
| M-PROMO7.1 | What system / criteria (e.g., product classification system or nutrient profiling system) does the company use to classify the healthiness of products for the purposes of **promotion to children**?  *(Note: check if the company supports the EU Pledge. If yes and no other comments, then EU pledge is scored)* | 10: Adopted an official classification system (developed by WHO, PAHO, national government, etc.)  5: Developed own system that has been validated and shows strong alignment with official classification systems / dietary guidelines, published in peer-reviewed literature  2.5: Developed own system that has been validated and shows alignment with official classification systems / dietary guidelines, not published in peer-reviewed literature  0: No information / poor alignment / does not have a system |
| M-PROMO7.2 | If a proprietary product classification system has been developed, which products, nutrients and food characteristics are covered, and what are the details? | [Information only, not to be scored] |
|  | **Policy position on marketing of unhealthy foods to children** | |
| M-PROMO8.1 | Does the company publish its policy position (in relation to government policy) on reducing the exposure of children and /or adolescents to, and the power of, the marketing of unhealthy foods? | 10: Yes, on own website  5: Yes, on industry association website  0: Not publicly available |
| M-PROMO8.2 | Does the company’s policy position support WHO’s position on government-led policy action related to reducing the exposure of children and adolescents to, and the power of, the marketing of unhealthy foods, as articulated in the WHO Global Action Plan for NCDs, the WHO European Food and Nutrition Action Plan and other key WHO documents (such as the Report of the Commission on Ending Childhood Obesity)?  *According to the World Health Assembly resolution WHA63.14, marketing policy should aim to reduce the impact on children of marketing of foods high in saturated fats, trans-fatty acids, free sugars or salt by reducing both exposure of children to, and power of, marketing of foods high in these nutrients, with uniform implementation across all implementing bodies. The policy should include settings where children gather. The government should be the key stakeholder in developing the policy including implementation, monitoring and evaluation, and enforcement systems should be in place including clear definitions of sanctions. Additional details available at:*  *http://apps.who.int/iris/bitstream/10665/44416/1/9789241500210_eng.pdf*  *Part of Objective 1 EU Action plan: Establish strong measures to reduce the overall impact on children of all forms of marketing of foods high in energy, saturated fat, trans fats, sugar or salt. Experience suggests that self-regulatory, voluntary approaches have loopholes and government leadership is required.* | 10: Strong support (e.g., includes support for government-led action of marketing to children and adolescents, related to power and exposure)  5: Weak support (e.g., includes support for government-led action of marketing to children or adolescents, related to either power or exposure,)  0: No details available  -5: Somewhat opposed (e.g., opposes government-led efforts to restrict some aspects of promotion to children / adolescents)  -10: Strongly opposed (e.g., opposes any actions to reduce promotion to children) |
|  | | |
| **M-ACCESS** | **Product accessibility** | **Maximum total points = 60** |
| M-ACCESS1 | Does the company make a commitment to address the price / affordability of its healthier products relative to its unhealthy products?  (if applicable, i.e. if company has both ‘healthy’ and ‘unhealthy’ products) | 10: Clear and specific targets for the whole business, published and applied in Europe or multiple European countries  7.5: Clear and specific targets for whole business, not published and applied  5: Broad commitment, published and applied in Europe or multiple European countries  2.5: Broad commitment, not published or applied  0: No commitment/ no information available to the research team |
| M-ACCESS2 | Does the company have a policy to increase the number/proportion of healthy products in the company’s portfolio? | 10: Clear and specific commitment to increase the proportion of healthy products across portfolio, published and applied in Europe or multiple European countries  7.5: Clear and specific commitment to increase the proportion of healthy products across portfolio, not published and applied  5: General commitment to increasing the number of healthy products across the portfolio, published, and applied in Europe or multiple European countries  2.5: General commitment to increasing the number of healthy products across the portfolio, not published or applied  0: No commitment / no information |
| M-ACCESS3 | Does the company make a clear and specific commitment to increase the availability of **healthy** products in settings? | 5: Yes, published and clear commitment for a range of key settings (including remote communities, schools, hospitals and community events)  2.5: Yes, not published or clear commitment for some specific settings (e.g, schools, remote communities, community events or hospitals)  1: Some commitment applicable to one setting (e.g., schools, remote communities, community events or hospitals)  0: No commitment / no information |
| M-ACCESS4 | Does the company make a clear and specific commitment to decrease the availability of **unhealthy** products in settings? | 5: Yes, published and clear commitment for a range of key settings (including remote communities, schools, hospitals and community events)  2.5: Yes, not published or clear commitment for some specific settings (e.g, schools, remote communities, community events or hospitals)  1: Some commitment applicable to one setting (e.g., schools, remote communities, community events or hospitals)  0: No commitment / no information |
|  | **Classification system** |  |
| M-ACCESS5.1 | What system / criteria (e.g., product classification system or nutrient profiling system) does the company use to classify the healthiness of products for the purposes of **food pricing, distribution and/or availability**? | 10: Adopted an official classification system (developed by WHO, PAHO, national government, etc.)  5: Developed own system that has been validated and shows strong alignment with official classification systems / dietary guidelines, published in peer-reviewed literature  2.5: Developed own system that has been validated and shows alignment with official classification systems / dietary guidelines, not published in peer-reviewed literature  0: No information / poor alignment / does not have a system |
| M-ACCESS5.2 | If a proprietary product classification system has been developed, which products, nutrients and food characteristics are covered, and what are the details? | [Information only, not to be scored] |
|  | **Policy position in relation to fiscal policies** | |
| M-ACCESS6.1 | Does the company publish its policy position (in relation to government policy) on fiscal policies to make healthier foods relatively cheaper and unhealthy foods relatively more expensive? | 10: Yes, on own website  5: Yes, on industry association website  0: Not publicly available |
| M-ACCESS6.2 | Does the company’s policy position support WHO’s position on fiscal policies to make healthier foods relatively cheaper and unhealthy foods relatively more expensive, as articulated in the WHO Global Action Plan for NCDs, the WHO European Food and Nutrition Action Plan and the Report of the Commission on Ending Childhood Obesity, Recommendation 1.2)?  *(ECHO Statement on Recommendation 1.2: Implement an effective tax on sugar-sweetened beverages.)*  *(Global Action Plan: consider economic tools that are justified by evidence, and may include taxes and subsidies, that create incentives for behaviours associated with improved health outcomes, improve the affordability and encourage consumption of healthier food products and discourage the consumption of less healthy options.)*  *(Part of Objective 1 EU Action plan: Consider the range of economic tools, including supply chain incentives, targeted subsidies and taxes, that could decrease or increase price, notably at point of purchase, and that could improve the affordability of a healthy diet and discourage the consumption of food products high in energy, saturated fats, trans fats, sugar or salt.)* | 10: Strong support (e.g., includes support for taxes on unhealthy foods, broadly defined, as well as subsidies for healthy foods)  5: Weak support (e.g., includes support for taxes on unhealthy foods, narrowly defined, or subsidies for healthy foods)  0: No details available  -10: Strongly opposed (e.g., opposes soft drinks tax or unhealthy foods tax OR both taxes) |
|  | | |
| **M-RELAT** | **Relationships with other organisations** | **Maximum total points = 90** |
| M-RELAT1 | Does the company publish details of the **professional organisations** (e.g., professional associations for nutrition or dietetics, physical activity or exercise organisations, medical organisations or societies, etc.) and/or scientific events (e.g., conferences) it funds or supports, including awards/prizes, making clear the nature of that support? | 10: Yes, information on European activity or activity in multiple European countries is publicly available (website or document) in a consolidated and cumulative form OR active declaration/policy stating no activity in this area (either publicly available or disclosed to INFORMAS team)  5: Yes, information is available, but is not consolidated and easy to locate OR information is available at the global level only OR comprehensive information about their activities in the area provided to the project team  0: No information available / provided |
| M-RELAT2 | Does the company publish details of the **external research** (e.g., conducted by individuals/groups/organisations) it funds or supports, including awards/prizes? | 10: Yes, information on European activity or activity in multiple European countries is publicly available (website or document) in a consolidated and cumulative form OR active declaration/policy stating no activity in this area (either publicly available or disclosed to INFORMAS team)  5: Yes, information is available, but is not consolidated and easy to locate OR information is available at the global level only OR comprehensive information about their activities in the area provided to the project team  0: No information available / provided |
| M-RELAT3 | **For philanthropic funding,** does the company publish details of the groups or organisations it funds or supports? | 10: Yes, information on European activity or activity in multiple European countries is publicly available (website or document) in a consolidated and cumulative form OR active declaration/policy stating no activity in this area (either publicly available or disclosed to INFORMAS team)  5: Yes, information is available, but is not consolidated and easy to locate OR information is available at the global level only OR comprehensive information about their activities in the area provided to the project team  0: No information available / provided |
| M-RELAT4.1 | Does the company publish details of the **nutrition education / healthy diet oriented programs** it funds or supports? | 10: Yes, information on European activity or activity in multiple European countries is publicly available (website or document) in a consolidated and cumulative form OR active declaration/policy stating no activity in this area (either publicly available or disclosed to INFORMAS team)  5: Yes, information is available, but is not consolidated and easy to locate OR information is available at the global level only OR comprehensive information about their activities in the area provided to the project team  0: No information available / provided |
| M-RELAT4.2 | **For nutrition education / health diet oriented programs**, does the company have a commitment to align programs to national or regional dietary guidelines? | [Information only, not to be scored] |
| M-RELAT5 | Does the company publish details of the **active lifestyle programs** (sports, physical activity) it funds or supports? | 10: Yes, information on European activity or activity in multiple European countries is publicly available (website or document) in a consolidated and cumulative form OR active declaration/policy stating no activity in this area (either publicly available or disclosed to INFORMAS team)  5: Yes, information is available, but is not consolidated and easy to locate OR information is available at the global level only OR comprehensive information about their activities in the area provided to the project team  0: No information available / provided |
| M-RELAT6 | Does the company publish details of its involvement in public-private partnerships and/or joint ventures with government organisations / agencies? (in addition to those covered as part of M-RELAT4.1 and M-RELAT5) | 10: Yes, information on European activity or activity in multiple European countries is publicly available (website or document) in a consolidated and cumulative form OR active declaration/policy stating no activity in this area (either publicly available or disclosed to INFORMAS team)  5: Yes, information is available, but is not consolidated and easy to locate OR information is available at the global level only OR comprehensive information about their activities in the area provided to the project team  0: No information available / provided |
| M-RELAT7 | Does the company publish details of its political donations?  (when not prohibited by government policy) | 10: Yes, information on European-level activity is publicly available (on a company website or document) OR declaration of no activity in this area  5: Yes, for specific European countries only OR actively declares no activity in some countries  0: No |
| M-RELAT8 | Does the company publish its membership / support for / ownership of industry associations, think tanks, interest groups, community organisations or other organisations that lobby in relation to population nutrition and/or obesity and NCD issues? | 10: Yes, information on European activity or activity in multiple European countries is publicly available (website or document) in a consolidated and cumulative form OR active declaration/policy stating no activity in this area (either publicly available or disclosed to INFORMAS team)  5: Yes, information is available, but is not consolidated and easy to locate OR information is available at the global level only OR comprehensive information about their activities in the area provided to the project team  0: No information available / provided |
| M-RELAT9 | Does the company make publicly available its submissions (or submissions with which the company is associated, such as through industry associations) to public consultations regarding relevant population nutrition policies? | 10: Yes, on company website or in a document that is publicly available or available upon request OR active declaration/policy stating no activity in this area (either publicly available or disclosed to INFORMAS team)  5: Yes, through industry association/European website/document  0: No |
| M-RELAT10 | Does the company have written policy and guidelines related to any of the above (funding or support for professional organisations, external research, philanthropic funding, nutrition education / healthy diet oriented programs, active lifestyle programs), including details of how it will be involved in these activities? | [Information only, not to be scored] |

## **Table S2 – Phase 1: Indicators and scoring criteria for supermarkets**

| **Indicator ID** | **Domain and indicator** | **Scoring** |
| --- | --- | --- |
| **S-STRAT** | **Corporate strategy** | **Maximum total points = 30** |
| S-STRAT1 | Does the supermarket have an overarching commitment to improving population nutrition and health articulated in strategic documents (e.g., corporate strategy document, corporate responsibility reports)? | 10: Yes, a specific commitment to improving population nutrition and health, at the European level or at the global level with reference to the European market or multiple European countries, publicly available in strategic documents  7.5: Yes, a specific global commitment to improving population nutrition and health, publicly available in strategic documents  5: Yes, a European- or global- level commitment, but not publicly-available, OR general reference to nutrition and health as part of general corporate strategy  0: No clear commitments to improving population nutrition and health |
| S-STRAT2 | Does the supermarkets commitment to improving population nutrition and health (where it exists) include specific objectives and targets for obesity and NCDs? | (Can be multiple, max of 10 points)  2: Contains specific, measurable, achievable, relevant and time bound (SMART) objectives and targets  2: Recognition or reference to relevant priorities set out in the WHO Global Action Plan for the Prevention and Control of NCDs 2013 - 2020, the WHO European Food and Nutrition Action Plan 2015 – 2020, the Sustainable Development Goals, or the WHO Report on Ending Childhood Obesity  2: Recognition or reference to relevant priorities in European policy documents relating to population nutrition and obesity/NCD prevention (e.g. A Strategy for Europe on Nutrition, Overweight and Obesity related health issues; EU Action Plan on Childhood Obesity 2014-2020; Horizon 2020; Fruit and vegetable regime)  2: Comprehensive in nature (e.g., includes three or more domains in this document, such as formulation, marketing and labelling)  2: Key Performance Indicators (KPIs) (and/or remuneration) of management linked to nutrition strategy/policy/targets |
| S-STRAT3 | Does the supermarket regularly publish details of its approach to population nutrition and health related to obesity and NCDs? | 10: Regular, publicly available reports including reporting against objectives and targets, a clear outlook of future plans and challenges, external verification / review, and that specifically refers to Europe or multiple European countries  7.5: Regular, publicly available global reports with no specific reference to Europe or European countries  5: Annual reports including some of the relevant information  2.5: Irregular reporting  0: None published |
| **S-FORM** | **Product formulation (own-brand products only)** | **Maximum total points = 95** |
| S-FORM1 | Does the supermarket publish a comprehensive set of commitments or objectives related to new product development and reformulating its existing products with respect to reducing the nutrients of concern and energy (salt/sodium, saturated fats, trans fats, added sugar and kilojoules/portion size)? | 10: Yes, specific European commitments/objectives or specific global commitments/objectives that include specific reference to multiple European countries, publicly available  7.5: Yes, specific global commitments/objectives that are publicly available  5: Has specific European commitments/objectives or specific global commitments/objectives with reference to multiple European countries, but not publicly available  2.5: Has European or global-level commitments/objectives in this area that are available publicly, but these commitments/objectives are vague and non-specific OR has global commitments/objectives but not publicly available  0: No commitment/ no policy information available to the research team |
| S-FORM2 | Is the supermarket a signatory to European and/or global industry initiatives on product reformulation or do they commit to voluntary programs on product reformulation?  *(e.g. IFBA commitments on reformulation)* | 5: Yes, and noted on company website / annual reports  2.5 Yes, but not noted on company website / annual reports (e.g. government/ NGO/ industry organisation’s website or disclosed directly to INFORMAS)  0: No |
|  | **Salt/sodium** (only assess where relevant to a company’s product portfolio and if not mandatory according to European government policy)  REMARK: In some EU countries there may be policies in place | |
| S-FORM3.1 | Has the supermarket set a target/targets or provided detailed evidence of having taken substantive action to reduce/reach lower levels of salt/sodium in products? | 10: Set SMART targets or provided detailed evidence of having taken significant action in all key categories/subcategories, published  5: Targets (not necessarily SMART) set or significant action taken in some key products/sub-categories/not published  2.5: General or vague commitment to reducing levels of salt/sodium in products, published or disclosed to INFORMAS team  0: No target / no information |
| S-FORM3.2 | When is the baseline year and target year?  What are the targets? | [Information only, not to be scored] |
|  | **Trans and saturated fats** (only assess where relevant to a company’s product portfolio and if not mandatory according to European government policy, not applicable to beverage industry)  REMARK: In some EU countries there may be policies in place, e.g. Denmark | |
| S-FORM4.1 | Has the supermarket set a target/targets or provided detailed evidence of having taken significant action to reduce artificial trans-fat added to products during the manufacturing process, and is it applicable to Europe?  *!Keep in mind possible upcoming EU regulation on max content of trans fats in foods (open for consultation)* | 10: Set a target or provided detailed evidence of having taken significant action to eliminate trans-fat in all relevant categories/subcategories, published  5: Set a target or taken significant action to eliminate/reduce in some relevant products/sub-categories/not published  2.5: General or vague commitment to reducing/eliminating use of trans fats in products, published or disclosed to INFORMAS team  0: No target / no information |
| S-FORM4.2 | When is the baseline year and target year?  What are the targets? | [Information only, not to be scored] |
| S-FORM5.1 | Has the supermarket set a target/targets or provided detailed evidence of having taken substantive action to reduce/reach lower levels of saturated fats, and is it applicable to Europe? | 10: Set SMART targets or provided detailed evidence of having taken significant action in all key categories/subcategories, published  5: Targets (not necessarily SMART) set or taken significant action in some key products/sub-categories/not published  2.5: General or vague commitment to reducing use of saturated fats in products, published or disclosed to INFORMAS team  0: No target / no information |
| S-FORM5.2 | When is the baseline year and target year?  What are the targets? | [Information only, not to be scored] |
|  | **Added sugars** (only assess where relevant to a company’s own-brand product portfolio and if not mandatory according to government policy) | |
| S-FORM6.1 | Has the supermarket set a target/targets or provided detailed evidence of having taken substantive action to reduce/reach lower levels of added sugars, and is it applicable to Europe? | 10: Set SMART targets or provided detailed evidence of having taken significant action in all key categories/subcategories, published  5: Targets (not necessarily SMART) set or taken significant action in some key products/sub-categories / not published  2.5: General or vague commitment to reducing use of added sugars in products, published or disclosed to INFORMAS team  0: No target / no information |
| S-FORM6.2 | When is the baseline year and target year?  What are the targets? | [Information only, not to be scored] |
|  | **Portion size (energy content)** (only assess where relevant to a company’s own-brand product portfolio and if not mandatory according to government policy) | |
| S-FORM7.1 | Does the supermarket have a target/targets or provided detailed evidence of having taken substantive action to reduce the portion size / energy content of single serve snacks, and is it applicable to Europe? | 10: Set SMART targets or provided detailed evidence of having taken significant action in all key categories/subcategories, published  5: Targets (not necessarily SMART) set or taken significant action in some key products/sub-categories / not published  2.5: General or vague commitment to reducing portion size / energy content in products, published or disclosed to INFORMAS team  0: No target / no information |
| S-FORM7.2 | When is the baseline year and target year?  What are the targets? | [Information only, not to be scored] |
|  | **Classification system** | |
| S-FORM8.1 | What system / criteria (e.g., product classification system or nutrient profiling system) does the supermarket use to classify the healthiness of products for the purposes of **own-brand product development / reformulation**? | 10: Uses government guidelines/government endorsed classification system (where available e.g. WHO Europe nutrient profile model)  7.5**: Publicly** available system, developed in consultation with experts and in line with government guidelines, published in peer reviewed literature  5: **Publicly** available system, developed in consultation with experts and in line with government guidelines (not published in peer reviewed literature)  2.5: **Publicly** available system with no details of development/alignment with government guidelines OR **not** **publicly** available but developed in consultation with experts and aligned with government guidelines  0: No information / poor alignment / does not have a system |
| S-FORM8.2 | If a proprietary product classification system has been developed, which products, nutrients and food characteristics are covered, and what are the details? | [Information only, not to be scored] |
|  | **Policy position on reformulation** | |
| S-FORM9.1 | Does the supermarket publish its policy position (in relation to government policy) on **own-brand product reformulation?** | 10: Yes, on own website  5: Yes, on industry association website  2.5: Policy position made available to INFORMAS team  0: Not publicly available |
| S-FORM9.2 | Does the supermarkets policy position support WHO’s position on **product reformulation** in relation to nutrients of concern, as articulated in the Global Action Plan for the Prevention and Control of NCDs 2013-2020 or the WHO European Food and Nutrition Action Plan 2015–2020? | 10: Support for government-led efforts to reformulate the food supply in relation to several nutrients of concern  5: Support for government-led efforts to reformulate the food supply in relation to only one nutrient of concern  0: No details available  -5: Somewhat opposed (e.g., opposes some aspects of implementation of government-led efforts to reformulate the food supply)  -10: Opposed to government-led efforts to reformulate the food supply in relation to nutrients of concern |
| **S-LABEL** | **Nutrition labelling** | **Maximum total points = 150** |
|  | **General nutrition labelling information** | |
| S-LABEL1 | Does the supermarket commit to provide on-pack information on *trans-*fat content **for own-brand products?** | 2.5: Yes, on all relevant products  1: Yes, on some products  0: No commitment / no information available to the research team  N/A: if commitment to eliminate use of all industrially produced trans-fat across portfolio |
| S-LABEL2 | Does the supermarket commit to provide on-pack information on added sugar content **for own-brand products?** | 2.5: Yes, on all relevant products  1: Yes, on some products  0: No commitment / no information available to the research team |
| S-LABEL3 | Does the supermarket provide nutrition information online **for own brand products**? | 10: Yes, comprehensive nutrition information (calories, sodium, saturated fat, total fat, sugar) for most (>80%) products, including on a per 100g/100ml basis  7.5: Yes, comprehensive nutrition information (calories, sodium, saturated fat, total fat, sugar) for most (>80%) products, OR comprehensive nutrition information for all products per serving only  5: Comprehensive nutrition information for some (>50%) products  2.5: Limited nutrition information (i.e. does not include calories, sodium, saturated fat, total fat or sugar) for some (>50%) items  0: <50% of products or no information |
| M-LABEL4 | Does the company have a policy to provide information on food composition to the EU commission, on request?  (if applicable, e.g., information has been requested by government) | 5: Yes, all products (published or not published)  2.5: Yes, some products  0: No policy / no information available to the research team |
|  | **Front of pack (FOP) nutrition labelling (own brand products only)** | |
| S-LABEL5.1 | **IF A VOLUNTARY GOVERNMENT-ENDORSED FOP NUTRITION LABELLING SCHEME EXISTS:**  Does the supermarket have a published commitment to rolling out a government endorsed FOP labelling system on **own brand** products?  (e.g. Nutri-Score, Traffic light) | 10: Yes, with implementation plan across all product categories (published or unpublished)  7.5: Yes, with implementation plan across a selection of product categories (published or unpublished)  5: Yes, but with no specific implementation plan (published or unpublished)  0: No commitment / no information available to the research team |
| S-LABEL5.2 | If the supermarket **does not commit to full implementation of a government endorsed FOP labelling system**, what FOP labelling system does the supermarket use?  Remark: Own FOP labelling systems do hamper the development and implementation of government endorsed FOP labelling systems | 10: Interpretive information (such as, stars, traffic lights, warning labels, etc.), applied across all product categories  7.5: Symbols or logos that indicate healthy products, applied across all product categories  5: Numeric information with % of recommended daily intake, applied across all product categories  2.5: Numeric information on levels of key nutrients, not showing % of recommended daily intake, applied across all product categories  0: No FOP labelling used  **DIVIDE POINTS IN HALF IF ONLY USED FOR SOME PRODUCTS / CATEGORIES**  **N/A if full implementation of government endorsed system** |
|  | **Policy position on front of pack nutrition labelling** | |
| S-LABEL6.1 | Does the supermarket publish its policy position (in relation to government policy) on front of pack nutrition labelling? | 10: Yes, on own website  5: Yes, on industry association website  2.5: Policy position made available to INFORMAS team  0: Not publicly available |
| S-LABEL6.2 | Does the supermarkets policy position (in relation to government policy) support WHO’s position on front of pack nutrition labelling, as articulated in the WHO Report of the Commission on Ending Childhood Obesity (Recommendation 1.7) or the WHO European Food and Nutrition Action Plan?  *(Statement on Recommendation 1.7: Implement interpretive front-of-pack labelling, supported by public education of both adults and children for nutrition literacy)*  *(EU Action plan: Increase consumer-friendly labelling by establishing easy-to-understand or interpretative front - of package labels that help consumers to identify healthier options)* | 10: Strong support (with a focus on “interpretive” to mean readily understandable and providing easy comparison of products e.g. using traffic light colours or stars to compare products for those with low nutritional literacy; broadly implemented)  5: Weak support (e.g., supports scheme that only provides limited interpretive information, such as % Daily Intake Guide for energy content only)  0: No details available  -5: Somewhat opposed (e.g., opposes some aspects of implementation of an interpretive scheme)  -10: Strongly opposed (e.g., opposes interpretive labelling) |
|  | **Health and nutrition claims** | |
| S-LABEL7 | Does the supermarket state that it will place a health claim on a product (or use a health claim as part of product advertising) only when the product is 'healthy'? | 10: Yes, commitment is published  5: Yes, commitment is not published  0: No commitment /no information available to the research team |
| S-LABEL8 | Does the supermarket state that it will place a nutrition claim on a product (or use a nutrition claim as part of product advertising) only when the product is 'healthy'? | 10: Yes, commitment is published  5: Yes, commitment is not published  0: No commitment / no information available to the research team |
| S-LABEL9.1 | What system / criteria (e.g., product classification system or nutrient profiling system) does the supermarket use to classify the healthiness of own-brand products for the purposes of **health and/or nutrition claims?** | 10: Adopted an official classification system (developed by WHO, PAHO, national government, etc.)  5: Developed own system that has been validated and shows strong alignment with official classification systems / dietary guidelines, published in peer-reviewed literature  2.5: Developed own system that has been validated and shows alignment with official classification systems / dietary guidelines, not published in peer-reviewed literature  0: No information / poor alignment / does not have a system |
| S-LABEL9.2 | If a proprietary product classification system has been developed, which products, nutrients and food characteristics are covered, and what are the details? | [Information only, not to be scored] |
|  | **Nutrition labelling in-store / in-store nutrition education** | |
| S-LABEL10 | Does the supermarket use shelf tags that provide summary nutrition information (apart from possible FOP labelling)?  *(e.g. Guiding Stars, Health Star Rating, nutritioniQ, NuVal)* | 10: Yes, labelling system used for **all** product categories, classification based on an official classification system (developed by WHO, PAHO, national government, etc.).  7.5: Yes, labelling system used for **some** product categories, classification based on an official classification system (developed by WHO, PAHO, national government, etc.).  5: Yes, labelling system used for **all** product categories, classification based on own system that has been validated and shows strong alignment with official classification systems / dietary guidelines, published in peer-reviewed literature.  2.5: Yes, labelling system used for **some** product categories, classification based on own system that has been validated and shows strong / moderate alignment with official classification systems / dietary guidelines, not published in peer-reviewed literature  0: No |
| S-LABEL11 | Does the supermarket provide summary nutrition information online?  *(e.g. Nutriscore, NuVal)* | 10: Yes, labelling system used for **all** product categories, classification based on an official classification system (developed by WHO, PAHO, national government, etc.).  7.5: Yes, labelling system used for **some** product categories, classification based on an official classification system (developed by WHO, PAHO, national government, etc.).  5: Yes, labelling system used for **all** product categories, classification based on own system that has been validated and shows strong alignment with official classification systems / dietary guidelines, published in peer-reviewed literature.  2.5: Yes, labelling system used for **some** product categories, classification based on own system that has been validated and shows strong / moderate alignment with official classification systems / dietary guidelines, not published in peer-reviewed literature  0: No |
| S-LABEL12 | Does the supermarket have an ongoing nutrition/healthy eating education program in-store? (e.g., dietitians in stores, nutrition education materials, etc.) | 5: Yes, in all/most stores  2.5: Seasonal /intermittent programs only, or only in some selected stores  1: Actively considering/engaged in options for nutrition/healthy eating education programs  0: No |
|  | **Menu labelling (if takeaway/ready to eat foods prepared on site; if not: Non-applicable)** | |
| S-LABEL13.1 | Does the supermarket commit to disclose nutrition information (e.g., on menus) for takeaway or ready-to-eat foods that are prepared on site? | 10: Yes, relates to all menu items and commitment is publicly available  7.5: Yes, relates to all menu items, commitment is not publicly available  5: Yes, relates to some menu items and commitment is publicly available  2.5: Yes, relates to some menu items, commitment is not publicly available  0: No commitment / no information available to the research team |
| S-LABEL13.2 | What nutrition information does the supermarket commit to providing (e.g., on menus) for takeaway or ready-to-eat foods that are prepared on site?  (only applicable if not government mandated and if these services are offered in store) | *Up to 10 points maximum:*  5: Energy / calories  5: Symbol or logo indicating ‘healthy’ items  2: Sodium/salt  2: Saturated fat  2: Total fat  2: Trans fat  2: Sugar |
| S-LABEL13.3 | If energy / calorie information is displayed, does the supermarket provide a contextual statement regarding the number of kJ / calories that should be consumed in a day for the average adult to maintain a healthy weight? | 5: Yes  0: No  N/A where mandated by government policy |
| S-LABEL14 | Does the supermarket publish its policy position (in relation to government policy if government policy exists) on **menu labelling?** | 10: Yes, on own website  5: Yes, on industry association website  2.5: Policy position made available to INFORMAS team  0: Not publicly available |
| **S-PROMO** | **Product and brand promotion** | **Maximum total points = 160** |
|  | **Promotion to children and adolescents– broadcast media** | |
| S-PROMO1.1 | Does the supermarket have an explicit policy to reduce the exposure of children to unhealthy food marketing on broadcast media (TV, radio)?  (*Note: check if the supermarket supports the EU Pledge. If yes and no other comments, then EU pledge is scored)* | 10: Yes, European policy or policy that refers to multiple European countries and noted on company website / annual reports  7.5: Yes, global policy and noted on company website / annual reports  5: Yes, European policy or policy that refers to multiple European countries, but not noted on company website / annual reports OR noted on industry association website  2.5: Yes, global policy but not noted on company website / annual reports  0: No policy/ no information available to the research team |
| S-PROMO1.2 | To what age group(s) does the broadcast marketing policy apply?  (*Note: check if the supermarket supports the EU Pledge. If yes and no other comments, then EU pledge is scored)* | 10: 18 years and under  8: 16 years and under  6: 14 years and under  4: 12 years and under  2: Under 10 years  0: No policy / no information |
| S-PROMO1.3 | How is the ‘target audience’ or ‘audience exposed’ defined?  (*Note: check if the supermarket supports the EU Pledge. If yes and no other comments, then EU pledge is scored)* | 10: Time-based restrictions, based on children’s peak viewing times (e.g., no advertising before 9:00pm)  5: Based on a percentage of the children’s audience that are likely to be viewing (e.g. if >10% of total children are watching)  2.5: Based on a percentage of the audience that are likely to be children (e.g., if >10% of audience are children)  1: Children’s programmes only  0: No explicit threshold / definition |
|  | **Promotion to children and adolescents – non-broadcast media** | |
| S-PROMO2.1 | Does the supermarket have an explicit policy to reduce the exposure of children to unhealthy food marketing on non-broadcast media (including websites, DVDs/games, social media, print media, product placement, outdoor marketing, in store marketing / point of sales marketing)?  (*Note: check if the supermarket supports the EU Pledge. If yes and no other comments, then EU pledge is scored)* | 10: Yes, European policy or policy that refers to multiple European countries and noted on company website / annual reports  7.5: Yes, global policy and noted on company website / annual reports  5: Yes, European policy or policy that refers to multiple European countries, but not noted on company website / annual reports OR noted on industry association website  2.5: Yes, global policy but not noted on company website / annual reports  0: No policy/ no information available to the research team  [Information only – what specific media channels are included] |
| S-PROMO2.2 | To what age group(s) does the non-broadcast marketing policy apply?  (*Note: check if the supermarket supports the EU Pledge. If yes and no other comments, then EU pledge is scored)* | 10: 18 years and under  8: 16 years and under  6: 14 years and under  4: 12 years and under  2: Under 10 years  0: No policy / no information |
|  | **Promotion to children and adolescents – general** | |
| S-PROMO3.1 | Does the supermarket pledge not to use celebrities in marketing of products to children other than those that meet the company’s healthy standard?  (*Note: check if the supermarket supports the EU Pledge. If yes and no other comments, then EU pledge is scored)* | 5: All forms of marketing  2.5: Some forms of marketing (e.g., excludes packaging) or applies only to those characters that appeal primarily to children  0: No policy / no information available to the research team |
| S-PROMO3.2 | Does the supermarket pledge not to use fantasy and animation characters with a strong appeal to children in marketing of products other than those that meet the company’s healthy standard?  (*Note: check if the supermarket supports the EU Pledge. If yes and no other comments, then EU pledge is scored)* | 5: All forms of marketing (includes no use of characters with strong appeal to children across **all** forms of marketing)  2.5: Some forms of marketing (includes no use of characters with strong appeal to children across **some** forms of marketing)  0: No policy / no information available to the research team |
| S-PROMO3.3 | Does the supermarket commit to not use premium offers (e.g., promotional toys, games, vouchers and competitions) in marketing of products other than those that meet the company’s healthy standard?  (*Note: check if the supermarket supports the EU Pledge. If yes and no other comments, then EU pledge is scored)* | 5: All forms of marketing  2.5: Some forms of marketing (e.g., excludes packaging)  0: No commitment / no information available to the research team |
| S-PROMO4 | Does the supermarket audit/monitor its compliance with its policy on marketing to children at the?  *(Note: check if the company supports the EU Pledge. If yes and no other comments, then EU pledge is scored)* | 5: Yes, the policy is audited by an independently appointed third party  2.5: Yes, the policy independently audited  1: No, the policy is not independently audited  0: No auditing is conducted |
|  | **Classification system** |  |
| S-PROMO5.1 | What system / criteria (e.g., product classification system or nutrient profiling system) does the supermarket use to classify the healthiness of products for the purposes of **promotion to children and adolescents**? | 10: Adopted an official classification system (developed by WHO, PAHO, national government, etc.)  5: Developed own system that has been validated and shows strong alignment with official classification systems / dietary guidelines, published in peer-reviewed literature  2.5: Developed own system that has been validated and shows alignment with official classification systems / dietary guidelines, not published in peer-reviewed literature  0: No information / poor alignment / does not have a system |
| S-PROMO5.2 | If a proprietary product classification system has been developed, which products, nutrients and food characteristics are covered, and what are the details? | [Information only, not to be scored] |
|  | **Policy position on marketing of unhealthy foods** | |
| S-PROMO6.1 | Does the supermarket publish its policy position (in relation to government policy) on reducing the exposure of children and /or adolescents to, and the power of, the marketing of unhealthy foods? | 10: Yes, on own website  5: Yes, on industry association website  0: Not publicly available |
| S-PROMO6.2 | Does the supermarkets policy position support WHO’s position on **government**-**led** policy action related to reducing the exposure of children and adolescents to, and the power of, the marketing of unhealthy foods, as articulated in the WHO Global Action Plan for NCDs, the WHO European Food and Nutrition Action Plan and other key WHO documents (such as the Report of the Commission on Ending Childhood Obesity)?  *According to the World Health Assembly resolution WHA63.14, marketing policy should aim to reduce the impact on children of marketing of foods high in saturated fats, trans-fatty acids, free sugars or salt by reducing both exposure of children to, and power of, marketing of foods high in these nutrients, with uniform implementation across all implementing bodies. The policy should include settings where children gather. The government should be the key stakeholder in developing the policy including implementation, monitoring and evaluation, and enforcement systems should be in place including clear definitions of sanctions. Additional details available at:*  *http://apps.who.int/iris/bitstream/10665/44416/1/9789241500210_eng.pdf*  *Part of Objective 1 EU Action plan: Establish strong measures to reduce the overall impact on children of all forms of marketing of foods high in energy, saturated fat, trans fats, sugar or salt. Experience suggests that self-regulatory, voluntary approaches have loopholes and government leadership is required.* | 10: Strong support (e.g., includes **support for government-led** action of marketing to children and adolescents, related to power and exposure)  5: Weak support (e.g., includes support for government-led action of marketing to children or adolescents, related to either power or exposure,)  0: No details available  -5: Somewhat opposed (e.g., opposes government-led efforts to restrict some aspects of promotion to children / adolescents)  -10: Strongly opposed (e.g., opposes any actions to reduce promotion to children) |
|  | **Promotion to all consumers** | |
| S-PROMO7 | Does the supermarket have a marketing policy to reduce the power and exposure of all consumers to unhealthy food marketing? | 5: Yes, and noted on company website / annual reports  2.5 Yes, but not noted on company website / annual reports  0: No policy / no information available to the research team |
| S-PROMO8 | To which media / settings does the marketing policy (related to all consumers) apply? | (Can be multiple)  5: Broadcast media (TV, Radio)  5: Non-broadcast media (including websites, DVDs/games, social media, print media, product placement, outdoor marketing) |
| S-PROMO9 | Does the supermarket have a policy to limit their in-store promotion of unhealthy products? | 10: Yes, published policy commits to only promote healthy products in-store  7.5: Yes, published policy commits to ensuring that a minimum proportion of in-store promotion is for healthy products  5: Policy exists, but not published  0: No policy / no information available to the research team |
| S-PROMO10 | Does the supermarket have a policy on the proportion of healthy (compared with unhealthy foods) foods promoted in their regular catalogues/circulars? | 10: Yes, published policy commits to only promote healthy products in regular catalogues/circulars  7.5: Yes, published policy commits to ensuring that a minimum proportion of products promoted in regular catalogues/circulars is for healthy products  5: Policy exists, but not published  0: No policy / no information available to the research team |
| S-PROMO11 | Does the supermarket have a policy to link rewards programs or loyalty programs to healthy food items? | 5: Yes, published policy commits to link rewards / loyalty schemes to healthy food products  2.5: Yes, published policy commits to link a proportion of rewards / loyalty schemes to healthy food products / not published  0: No policy / no information available to the research team |
| S-PROMO12 | Does the supermarket have a policy to ensure that in-store product presentations, product giveaways or tastings are for healthy products (including giveaways to children)? | 5: All presentations, product giveaways or tastings must be for healthy products  2.5: Some presentations or tastings (e.g. those aimed at children) must be for healthy products  0: No policy / no information available to the research team |
| S-PROMO13 | Does the supermarket audit/monitor its compliance with its policy on marketing to all consumers? | 5: Yes, the policy is audited by an independently appointed third party  2.5: Yes, the policy independently audited  1: No, the policy is not independently audited  0: No auditing is conducted |
|  | **Product classification for promotion to all consumers (including children)** | |
| S-PROMO14.1 | What system / criteria (e.g., product classification system or nutrient profiling system) does the supermarket use to classify the healthiness of products for the purposes of **product promotion to all consumers (e.g. in-store catalogues, brochures, flyers, shelf tags, promotional posters)**? | 10: Adopted an official classification system (developed by WHO, PAHO, national government, etc.)  5: Developed own system that has been validated and shows strong alignment with official classification systems / dietary guidelines, published in peer-reviewed literature  2.5: Developed own system that has been validated and shows alignment with official classification systems / dietary guidelines, not published in peer-reviewed literature  0: No information / poor alignment / does not have a system |
| S-PROMO14.2 | If a proprietary product classification system has been developed, which products, nutrients and food characteristics are covered, and what are the details? | [Information only, not to be scored] |
| **S-ACCESS** | **Product accessibility** | **Maximum total points = 120** |
| S-ACCESS1 | Does the supermarket make a commitment to address the price / affordability of healthy products relative to unhealthy products, particularly where there are comparable substitutes? | 10: Commitment that standard prices of healthy products are lower than standard prices of comparable unhealthy products  7.5: Commitment that standard prices of **own-brand** healthy products are lower than standard prices of comparable **own-brand** unhealthy products  5: Commitment that standard prices of healthy products are equivalent to standard prices of comparable unhealthy products  2.5: Commitment that standard prices **own-brand** healthy products are equivalent to standard prices of comparable **own-brand** unhealthy products OR broad commitment only (related to own-brand products or other products)  0: No commitment / no information available to the research team |
| S-ACCESS2 | Does the supermarket have a published position on the size and nature of discounts / price promotions applied to healthy and unhealthy foods? | 10: Commitment to no price promotions on unhealthy foods  7.5: Commitment to greater levels of discount typically applying to healthy foods compared to unhealthy foods across all food categories  5: Commitment to the same types / levels of discounts typically applying on healthy and unhealthy foods across all food categories  2.5: Commitment to the same types / levels of discounts typically applying on healthy and unhealthy foods for some food categories  0: No policy / no information available to the research team |
| S-ACCESS3 | Does the supermarket make a commitment to limit multi-buy specials (e.g. two for one) on unhealthy foods? | 10: Commitment to no multi-buy specials for unhealthy foods  5: Commitment to limit multi-buy specials for unhealthy foods  0: No commitment / no information available to the research team |
|  | **Policy position in relation to fiscal policies** | |
| S-ACCESS4.1 | Does the supermarket publish its policy position (in relation to government policy) on fiscal policies to make healthier foods relatively cheaper and unhealthy foods relatively more expensive? | 10: Yes, on own website  5: Yes, on industry association website  0: Not publicly available |
| S-ACCESS4.2 | Does the supermarkets policy position support WHO’s position on fiscal policies to make healthier foods relatively cheaper and unhealthy foods relatively more expensive, as articulated in the WHO Global Action Plan for NCDs, the WHO European Food and Nutrition Action Plan and other key WHO documents (such as the Report of the Commission on Ending Childhood Obesity, Recommendation 1.2)?  *(ECHO Statement on Recommendation 1.2: Implement an effective tax on sugar-sweetened beverages.)*  *(Global Action Plan: consider economic tools that are justified by evidence, and may include taxes and subsidies, that create incentives for behaviours associated with improved health outcomes, improve the affordability and encourage consumption of healthier food products and discourage the consumption of less healthy options.)*  *(Part of Objective 1 EU Action plan: Consider the range of economic tools, including supply chain incentives, targeted subsidies and taxes, that could decrease or increase price, notably at point of purchase, and that could improve the affordability of a healthy diet and discourage the consumption of food products high in energy, saturated fats, trans fats, sugar or salt.)* | 10: Strong support (e.g., includes support for taxes on unhealthy foods or sugar sweetened beverages, broadly defined, as well as subsidies for healthy foods)  5: Weak support (e.g., includes support for taxes on unhealthy foods or sugar sweetened beverages, narrowly defined, or subsidies for healthy foods)  0: No details available  -5: Somewhat opposed (e.g., opposes taxes on unhealthy foods or subsidies for healthy foods)  -10: Strongly opposed (e.g., opposes all measures in this area) |
|  | **Distribution/availability (own-brand products)** | |
| S-ACCESS5.1 | Does the supermarket have a policy to increase the number/proportion of healthy products in the supermarkets own portfolio? | 10: Clear and specific commitment to increase the proportion of healthy products across portfolio, published and applicable to Europe or multiple European countries.  7.5: Clear and specific commitment to increase the proportion of healthy products across portfolio, not published, but applicable to Europe or multiple European countries.  5: General commitment to increasing the number of healthy products across the portfolio, published and applicable to Europe, in multiple European countries or globally  2.5: General commitment to increasing the number of healthy products across the portfolio, not published  0: No commitment / no information |
| S-ACCESS5.2 | Does the supermarket make a clear and specific commitment to increase the availability of **healthy** products in settings? | 5: Yes, published and clear commitment for a range of key settings (including remote communities, schools, hospitals and community events)  2.5: Yes, not published or clear commitment for some specific settings (e.g, schools, remote communities, community events or hospitals)  1: Some commitment applicable to one setting (e.g., schools, remote communities, community events or hospitals)  0: No commitment / no information |
| S-ACCESS5.3 | Does the supermarket make a clear and specific commitment to decrease the availability of **unhealthy** products in settings? | 5: Yes, published and clear commitment for a range of key settings (including remote communities, schools, hospitals and community events)  2.5: Yes, not published or clear commitment for some specific settings (e.g, schools, remote communities, community events or hospitals)  1: Some commitment applicable to one setting (e.g., schools, remote communities, community events or hospitals)  0: No commitment / no information |
|  | **In-store availability initiatives** | |
| S-ACCESS6 | Does the supermarket make a clear and specific commitment to dedicate a minimum amount or proportion of shelf space or floor space to healthy products? | 10: Clear commitment for whole business, and is published  7.5: Clear commitment for whole business, is not published  5: Broad commitment, is published  2.5: Broad commitment, is not published  0: No commitment / no information available to the research team |
| S-ACCESS7 | Does the supermarket make a clear and specific commitment to dedicate a maximum amount or proportion of shelf space or floor space to less healthy products? | 10: Clear commitment for whole business, and is published  7.5: Clear commitment for whole business, is not published  5: Broad commitment, is published  2.5: Broad commitment, is not published  0: No commitment / no information available to the research team |
| S-ACCESS8 | Does the supermarket have a policy that checkouts are free from unhealthy items (including confectionery, chocolate and soft drinks)?  *(No unhealthy items displayed near the cash register)* | 10: Yes, no unhealthy items, applies to all checkouts in stores across Europe  7.5: Yes, no unhealthy items, applies to some checkouts OR limit unhealthy items, applies to all checkouts (in stores across Europe)  5: Limit unhealthy items, applies to some checkouts OR applies to stores in some countries in Europe  2.5: Actively considering/engaged in healthy checkout options  0: No policy / no information available to the research team |
| S-ACCESS9 | Does the supermarket have a published position on the placement of unhealthy items (such as confectionery, chocolate and soft drinks) at end of aisle displays or other high-traffic areas? | 10: Yes, no unhealthy items, applies to all high-traffic areas  7.5: Yes, no unhealthy items, applies to some high-traffic areas  5: Limit unhealthy items, applies to all high-traffic areas  2.5: Limit unhealthy items, applies to some high-traffic areas  0: No commitment / no information available to the research team |
|  | **Product classification** |  |
| S-ACCESS10.1 | What system / criteria (e.g., product classification system or nutrient profiling system) does the supermarket use to classify the healthiness of products for the purposes of **product pricing and availability (e.g. dedicated amount of shelf space, product placement at end of aisles/high traffic areas, product placement at checkout)**? | 10: Adopted an official classification system (developed by WHO, PAHO, national government, etc.)  5: Developed own system that has been validated and shows strong alignment with official classification systems / dietary guidelines, published in peer-reviewed literature  2.5: Developed own system that has been validated and shows alignment with official classification systems / dietary guidelines, not published in peer-reviewed literature  0: No information / poor alignment / does not have a system |
| S-ACCESS10.2 | If a proprietary product classification system has been developed, which products, nutrients and food characteristics are covered, and what are the details? | [Information only, not to be scored] |
| **S-RELAT** | **Relationships with other organisations** | **Maximum total points = 90** |
| S-RELAT1 | Does the supermarket publish details of the **professional organisations** (e.g., professional associations for nutrition or dietetics, physical activity or exercise organisations, medical organisations or societies, etc.) and/or scientific events (e.g., conferences) it funds or supports, including awards/prizes, making clear the nature of that support? | 10: Yes, information on European activity or activity in multiple European countries is publicly available (website or document) in a consolidated and cumulative form OR active declaration/policy stating no activity in this area (either publicly available or disclosed to INFORMAS team)  5: Yes, information is available, but is not consolidated and easy to locate OR information is available at the global level only OR comprehensive information about their activities in the area provided to the project team  0: No information available / provided |
| S-RELAT2 | Does the supermarket publish details of the **external research** (e.g., conducted by individuals/groups/organisations) it funds or supports, including awards/prizes? | 10: Yes, information on European activity or activity in multiple European countries is publicly available (website or document) in a consolidated and cumulative form OR active declaration/policy stating no activity in this area (either publicly available or disclosed to INFORMAS team)  5: Yes, information is available, but is not consolidated and easy to locate OR information is available at the global level only OR comprehensive information about their activities in the area provided to the project team  0: No information available / provided |
| S-RELAT3 | **For philanthropic funding,** does the supermarket publish details of the groups or organisations it funds or supports? | 10: Yes, information on European activity or activity in multiple European countries is publicly available (website or document) in a consolidated and cumulative form OR active declaration/policy stating no activity in this area (either publicly available or disclosed to INFORMAS team)  5: Yes, information is available, but is not consolidated and easy to locate OR information is available at the global level only OR comprehensive information about their activities in the area provided to the project team  0: No information available / provided |
| S-RELAT4.1 | Does the supermarket publish details of the **nutrition education / healthy diet oriented programs** it funds or supports? | 10: Yes, information on European activity or activity in multiple European countries is publicly available (website or document) in a consolidated and cumulative form OR active declaration/policy stating no activity in this area (either publicly available or disclosed to INFORMAS team)  5: Yes, information is available, but is not consolidated and easy to locate OR information is available at the global level only OR comprehensive information about their activities in the area provided to the project team  0: No information available / provided |
| S-RELAT4.2 | **For nutrition education / health diet oriented programs**, does the supermarket have a commitment to align programs to national or regional dietary guidelines? | [Information only, not to be scored] |
| S-RELAT5 | Does the supermarket publish details of the **active lifestyle programs** (sports, physical activity) it funds or supports? | 10: Yes, information on European activity or activity in multiple European countries is publicly available (website or document) in a consolidated and cumulative form OR active declaration/policy stating no activity in this area (either publicly available or disclosed to INFORMAS team)  5: Yes, information is available, but is not consolidated and easy to locate OR information is available at the global level only OR comprehensive information about their activities in the area provided to the project team  0: No information available / provided |
| S-RELAT6 | Does the supermarket publish details of its involvement in public-private partnerships and/or joint ventures with government organisations / agencies? (in addition to those covered as part of S-RELAT4.1 and S-RELAT5) | 10: Yes, information on European activity or activity in multiple European countries is publicly available (website or document) in a consolidated and cumulative form OR active declaration/policy stating no activity in this area (either publicly available or disclosed to INFORMAS team)  5: Yes, information is available, but is not consolidated and easy to locate OR information is available at the global level only OR comprehensive information about their activities in the area provided to the project team  0: No information available / provided |
| S-RELAT7 | Does the supermarket publish details of its political donations?  (when not prohibited by government policy) | 10: Yes, information on European-level activity is publicly available (on a company website or document) OR declaration of no activity in this area  5: Yes, for specific European countries only OR actively declares no activity in some countries  0: No |
| S-RELAT8 | Does the supermarket publish its membership / support for / ownership of industry associations, think tanks, interest groups, community organisations or other organisations that lobby in relation to population nutrition and/or obesity and NCD issues? | 10: Yes, information on European activity or activity in multiple European countries is publicly available (website or document) in a consolidated and cumulative form OR active declaration/policy stating no activity in this area (either publicly available or disclosed to INFORMAS team)  5: Yes, information is available, but is not consolidated and easy to locate OR information is available at the global level only OR comprehensive information about their activities in the area provided to the project team  0: No information available / provided |
| M-RELAT9 | Does the company make publicly available its submissions (or submissions with which the company is associated, such as through industry associations) to public consultations regarding relevant population nutrition policies? | 10: Yes, on company website or in a document that is publicly available or available upon request OR active declaration/policy stating no activity in this area (either publicly available or disclosed to INFORMAS team)  5: Yes, through industry association/European website/document  0: No |
| S-RELAT10 | Does the company have written policy and guidelines related to any of the above (funding or support for professional organisations, external research, philanthropic funding, nutrition education / healthy diet oriented programs, active lifestyle programs), including details of how it will be involved in these activities? | [Information only, not to be scored] |

## **Table S3 – Phase 1: Indicators and scoring criteria for chain restaurants**

| **Indicator ID** | **Domain and indicator** | **Scoring** |
| --- | --- | --- |
| **R-STRAT** | **Corporate strategy** | **Maximum total points = 30** |
| R-STRAT1 | Does the chain restaurant have an overarching commitment to improving population nutrition and health articulated in strategic documents (e.g., corporate strategy document, corporate responsibility reports)? | 10: Yes, a specific commitment to improving population nutrition and health, at the European level or at the global level with reference to the European market or multiple European countries, publicly available in strategic documents  7.5: Yes, a specific global commitment to improving population nutrition and health, publicly available in strategic documents  5: Yes, a European- or global- level commitment, but not publicly-available, OR general reference to nutrition and health as part of general corporate strategy  0: No clear commitments to improving population nutrition and health |
| R-STRAT2 | Does the chain restaurants commitment to improving population nutrition and health (where it exists) include specific objectives and targets for obesity and NCDs? | (Can be multiple, max of 10 points)  2: Contains specific, measurable, achievable, relevant and time bound (SMART) objectives and targets  2: Recognition or reference to relevant priorities set out in the WHO Global Action Plan for the Prevention and Control of NCDs 2013 - 2020, the WHO European Food and Nutrition Action Plan 2015 – 2020, the Sustainable Development Goals, or the WHO Report on Ending Childhood Obesity  2: Recognition or reference to relevant priorities in European policy documents relating to population nutrition and obesity/NCD prevention (e.g. A Strategy for Europe on Nutrition, Overweight and Obesity related health issues; EU Action Plan on Childhood Obesity 2014-2020; Horizon 2020; Fruit and vegetable regime)  2: Comprehensive in nature (e.g., includes three or more domains in this document, such as formulation, marketing and labelling)  2: Key Performance Indicators (KPIs) (and/or remuneration) of management linked to nutrition strategy/policy/targets |
| R-STRAT3 | Does the chain restaurant regularly publish details of its approach to population nutrition and health related to obesity and NCDs? | 10: Regular, publicly available reports including reporting against objectives and targets, a clear outlook of future plans and challenges, external verification / review, and that specifically refers to Europe or multiple European countries  7.5: Regular, publicly available global reports with no specific reference to Europe or European countries  5: Regular reports including some of the relevant information  2.5: Irregular reporting  0: None published |
| **R-FORM** | **Product formulation** | **Maximum total points = 100** |
| R-FORM1 | Does the chain restaurant publish a comprehensive set of commitments or objectives related to new product development and reformulating its existing products with respect to nutrients of concern and energy (salt/sodium, saturated fats, trans fats, added sugar and kilojoules/portion sizes)? | 10: Yes, specific European commitments/objectives or specific global commitments/objectives that include specific reference to multiple European countries, publicly available  7.5: Yes, specific global commitments/objectives that are publicly available  5: Has specific European commitments/objectives or specific global commitments/objectives with reference to multiple European countries, but not publicly available  2.5: Has European or global-level commitments/objectives in this area that are available publicly, but these commitments/objectives are vague and non-specific OR has global commitments/objectives but not publicly available  0: No commitment/ no policy information available to the research team |
| R-FORM2 | Is the chain restaurant a signatory to European and/or global industry initiatives on product reformulation or do they commit to voluntary programs on product reformulation?  *(e.g. IFBA commitments on reformulation)* | 5: Yes, and noted on company website / annual reports  2.5 Yes, but not noted on company website / annual reports (e.g. government/ NGO/ industry organisation’s website or disclosed directly to INFORMAS)  0: No / no information |
|  | **Salt/sodium** (only assess where relevant to a company’s product portfolio and if not mandatory according to European government policy)  REMARK: In some EU countries there may be policies in place | |
| R-FORM3.1 | Has the chain restaurant set a target/targets or provided detailed evidence of having taken substantive action to reduce/reach lower levels of salt/sodium in products, and is it applicable to Europe? | (Can be multiple)  5: Set SMART target or provided detailed evidence of having taken substantive action for children’s meals, published (2.5 if not published, or not SMART)  5: Set SMART target or provided detailed evidence of having taken substantive action for other relevant products/sub-categories, published (2.5 if not published, or not SMART)  0: No target / no information |
| R-FORM3.2 | When is the baseline year and target year?  What are the targets? | [Information only, not to be scored] |
|  | **Trans and saturated fats** (only assess where relevant to a company’s product portfolio and if not mandatory according to European government policy, not applicable to beverage industry)  REMARK: In some EU countries there may be policies in place, e.g. Denmark | |
| R-FORM4.1 | Has the chain restaurant set a target/targets or provided detailed evidence of having taken significant action to reduce artificial trans-fat added to products during the manufacturing process, and is it applicable to Europe?  *!Keep in mind possible upcoming EU regulation on max content of trans fats in foods (open for consultation)* | 10: Set a target or provided detailed evidence of having taken significant action to eliminate trans fat in all relevant categories/subcategories, published  5: Set a target or taken significant action to eliminate/reduce in some relevant products/sub-categories/not published  2.5: General or vague commitment to reducing/eliminating use of trans fats in products, published or disclosed to INFORMAS team  0: No target / no information |
| R-FORM4.2 | When is the baseline year and target year?  What are the targets? | [Information only, for evidence document, not to be scored] |
| R-FORM5.1 | Has the chain restaurant set a target/targets or provided detailed evidence of having taken substantive action to reduce/reach lower levels of saturated fats, and is it applicable to Europe? | (Can be multiple)  5: Set SMART target or provided detailed evidence of having taken substantive action for children’s meals, published (2.5 if not published, or not SMART)  5: Set SMART target or provided detailed evidence of having taken substantive action for other relevant products/sub-categories, published (2.5 if not published, or not SMART)  0: No target / no information |
| R-FORM5.2 | When is the baseline year and target year?  What are the targets? | [Information only, for evidence document, not to be scored] |
| R-FORM5.3 | Does the chain restaurant have commitments or taken substantive action to improving the healthiness of oils used in frying foods / frying practices? (if applicable) | 5: Does not fry foods, or commits to using non-hydrogenated vegetable oils  0: No commitments / no information available to the research team |
|  | **Added sugars** (only assess where relevant to a company’s product portfolio and if not mandatory according to government policy) | |
| R-FORM6.1 | Has the chain restaurant set a target/targets or provided detailed evidence of having taken substantive action to reduce/reach lower levels of added sugars, and is it applicable to Europe? | (Can be multiple)  5: Set SMART target or provided detailed evidence of having taken substantive action for children’s meals, published (2.5 if not published, or not SMART)  5: Set SMART target or provided detailed evidence of having taken substantive action for other relevant products/sub-categories, published (2.5 if not published, or not SMART)  0: No target / no information |
| R-FORM6.2 | When is the baseline year and target year?  What are the targets? | [Information only, for evidence document, not to be scored] |
|  | **Portion size (energy content)** (only assess where relevant to a company’s product portfolio and if not mandatory according to government policy) | |
| R-FORM7.1 | Does the chain restaurant have a target/targets or provided detailed evidence of having taken substantive action to reduce portion size / energy content, and is it applicable to Europe? | (Can be multiple)  5: Set SMART target or provided detailed evidence of having taken substantive action for children’s meals, published (2.5 if not published, or not SMART)  5: Set SMART target or provided detailed evidence of having taken substantive action for other relevant products/sub-categories, published (2.5 if not published, or not SMART)  0: No target / no information |
| R-FORM7.2 | When is the baseline year and target year?  What are the targets? | [Information only, for evidence document, not to be scored] |
|  | **Classification system** |  |
| R-FORM8.1 | What system / criteria (e.g., product classification system or nutrient profiling system) does the chain restaurant use to classify the healthiness of products **for the purposes of product development / reformulation**? | 10: Uses government guidelines/government endorsed classification system (where available e.g. WHO Europe nutrient profile model)  7.5**: Publicly** available system, developed in consultation with experts and in line with government guidelines, published in peer reviewed literature  5: **Publicly** available system, developed in consultation with experts and in line with government guidelines (not published in peer reviewed literature)  2.5: **Publicly** available system with no details of development/alignment with government guidelines OR **not** **publicly** available but developed in consultation with experts and aligned with government guidelines  0: No information / poor alignment / does not have a system |
| R-FORM8.2 | If a proprietary product classification system has been developed, which products, nutrients and food characteristics are covered, and what are the details? | [Information only, not to be scored] |
|  | **Policy position on reformulation** | |
| R-FORM9.1 | Does the chain restaurant publish its policy position (in relation to government policy) on **product reformulation?** | 10: Yes, on own website  5: Yes, on industry association website  0: Not publicly available |
| R-FORM9.2 | Does the chain restaurants policy position support WHO’s position on **product reformulation** in relation to nutrients of concern, as articulated in the Global Action Plan for the Prevention and Control of NCDs 2013-2020 or the WHO European Food and Nutrition Action Plan 2015 – 2020? | 10: Support for government-led efforts to reformulate the food supply in relation to several nutrients of concern  5: Support for government-led efforts to reformulate the food supply in relation to only one nutrient of concern  0: No details available  -5: Somewhat opposed (e.g., opposes some aspects of implementation of government-led efforts to reformulate the food supply)  -10: Opposed to government-led efforts to reformulate the food supply in relation to nutrients of concern |
| **R-LABEL** | **Nutrition labelling** | **Maximum total points = 60** |
| R-LABEL1 | Does the chain restaurant commit to disclose nutrition information on its menus? | 10: Yes, relates to all menu items and commitment is publicly available  7.5: Yes, relates to all menu items, commitment is not publicly available  5: Yes, relates to some menu items and commitment is publicly available  2.5: Yes, relates to some menu items, commitment is not publicly available  0: No commitment / no information available to the research team |
| R-LABEL2.1 | What nutrition information does the chain restaurant commit to providing on menus? | *Up to 10 points maximum:*  5: Energy / calories  5: Symbol or logo indicating ‘healthy’ items according to overall nutritional profile  2: Sodium/salt  2: Saturated fat  2: Total fat  2: Trans fat  2: Sugar |
| R-LABEL2.2 | If energy / calorie information is displayed on menus, does the chain restaurant provide a contextual statement regarding the number of calories that should be consumed in a day for the average adult to maintain a healthy weight? | 5: Yes  0: No / no information  N/A where mandated by government policy |
| R-LABEL3 | Does the chain restaurant provide nutrition information online? | 10: Yes, comprehensive nutrition information (calories, sodium, saturated fat, total fat, sugar) for most (>80%) products, including on a per 100g/100ml basis  7.5: Yes, comprehensive nutrition information (calories, sodium, saturated fat, total fat, sugar) for most (>80%) products, or comprehensive nutrition information for all products per serving only  5: Comprehensive nutrition information for some (>50%) products  2.5: Limited nutrition information (i.e. does not include calories, sodium, saturated fat, total fat or sugar) for some (>50%) products  0: <50% of products or no information |
| R-LABEL4 | Does the chain restaurant have a policy that they will provide comprehensive nutrition information in-store? | 10: Yes, comprehensive nutrition information (calories, sodium, saturated fat, total fat, sugar) for most (>80%) products, including on a per 100g/100ml basis  7.5: Yes, comprehensive nutrition information (calories, sodium, saturated fat, total fat, sugar) for most (>80%) products, or comprehensive nutrition information for all products per serving only  5: Comprehensive nutrition information for some (>50%) products  2.5: Limited nutrition information (i.e. does not include calories, sodium, saturated fat, total fat or sugar) for some (>50%) products  0: <50% of products or no information |
| R-LABEL5 | Does the chain restaurant have a policy to provide information on food composition to the EU commission, on request?  (if applicable, e.g., information has been requested by government) | 5: Yes, all products (published or not published)  2.5: Yes, some products  0: No policy / no information available to the research team |
| R-LABEL6 | Does the chain restaurant publish its policy position (in relation to government policy) on **menu labelling?** | 10: Yes, on own website  5: Yes, on industry association website  2.5: Policy position made available to INFORMAS team  0: Not publicly available |
| **R-PROMO** | **Product and brand promotion** | **Maximum total points = 130** |
|  | **Broadcast media** | |
| R-PROMO1.1 | Does the chain restaurant have an explicit policy to reduce the exposure of children to unhealthy food marketing on broadcast media (TV, radio)?  *(Note: check if the company supports the EU Pledge. If yes and no other comments, then EU pledge is scored)* | 10: Yes, European policy or policy that refers to multiple European countries and noted on company website / annual reports  7.5: Yes, global policy and noted on company website / annual reports  5: Yes, European policy or policy that refers to multiple European countries, but not noted on company website / annual reports OR noted on industry association website  2.5: Yes, global policy but not noted on company website / annual reports  0: No policy/ no information available to the research team |
| R-PROMO1.2 | To what age group(s) does the broadcast marketing policy apply?  *(Note: check if the company supports the EU Pledge. If yes and no other comments, then EU pledge is scored)* | 10: 18 years and under  8: 16 years and under  6: 14 years and under  4: 12 years and under  2: Under 10 years  0: No policy / no information |
| R-PROMO1.3 | How is the ‘target audience’ or ‘audience exposed’ defined?  *(Note: check if the company supports the EU Pledge. If yes and no other comments, then EU pledge is scored)* | 10: Time-based restrictions, based on children’s peak viewing times (e.g., no advertising before 9:00pm)  5: Based on a percentage of the children’s audience that are likely to be viewing (e.g. if >10% of total children are watching)  2.5: Based on a percentage of the audience that are likely to be children (e.g., if >10% of audience are children)  1: Children’s programmes only  0: No explicit threshold / definition |
|  | **Non-broadcast media** | |
| R-PROMO2.1 | Does the chain restaurant have an explicit policy to reduce the exposure of children to unhealthy food marketing on non-broadcast media (including websites, DVDs/games, social media, print media, product placement, outdoor marketing, in store marketing / point of sales marketing)?  *(Note: check if the company supports the EU Pledge. If yes and no other comments, then EU pledge is scored)* | 10: Yes, European policy or policy that refers to multiple European countries and noted on company website / annual reports  7.5: Yes, global policy and noted on company website / annual reports  5: Yes, European policy or policy that refers to multiple European countries, but not noted on company website / annual reports OR noted on industry association website  2.5: Yes, global policy but not noted on company website / annual reports  0: No policy/ no information available to the research team  [Information only – what specific media channels are included] |
| R-PROMO2.2 | To what age group(s) does the non-broadcast marketing policy apply?  *(Note: check if the company supports the EU Pledge. If yes and no other comments, then EU pledge is scored)* | 10: 18 years and under  8: 16 years and under  6: 14 years and under  4: 12 years and under  2: Under 10 years  0: No policy / no information |
| R-PROMO3 | Does the chain restaurant commit not to sponsor children’s sporting, cultural or other activities using unhealthy brands (foods or company brands)?  *(Note: check if the company supports the EU Pledge. If yes and no other comments, then EU pledge is scored)* | 10: Yes, comprehensive commitment including products and brands  5: Yes, comprehensive commitment including products only (brands still permitted)  2.5: Some commitments in the area, including some events or some forms of sponsorship  0: No commitment / no information available to the research team |
| R-PROMO4 | Does the chain restaurant commit not to use marketing in settings where children gather using unhealthy brands (foods or company brands)?  *(Note: check if the company supports the EU Pledge. If yes and no other comments, then EU pledge is scored)* | (Can be multiple)  2: Commits IN early childcare settings and primary schools (children up to age 11)  2: Commits NEAR (e.g. within 500m) of early childcare settings and primary schools (children up to age 11)  2: Commits IN secondary schools (children between age 12 and 18)  2: Commits NEAR (e.g., within 500m) of secondary schools (children between age 12 and 18)  2: Commits in other places where children gather (family and child clinics, paediatric services or other health facilities, sporting or recreation centres, or sporting or cultural events held at those premises) |
|  | **General policies to reduce promotion to children** | |
| R-PROMO5.1 | Does the chain restaurant pledge not to use celebrities in marketing of products other than those that meet the chain restaurants healthy standard?  *(Note: check if the company supports the EU Pledge. If yes and no other comments, then EU pledge is scored)* | 5: All forms of marketing  2.5: Some forms of marketing (e.g., excludes packaging) or applies only to those characters that appeal primarily to children  0: No / no information |
| R-PROMO5.2 | Does the chain restaurant pledge not to use fantasy and animation characters with a strong appeal to children in marketing of products other than those that meet the chain restaurants healthy standard?  *(Note: check if the company supports the EU Pledge. If yes and no other comments, then EU pledge is scored)* | 5: All forms of marketing (includes no use of characters with strong appeal to children across **all** forms of marketing)  2.5: Some forms of marketing (includes no use of characters with strong appeal to children across **some** forms of marketing)  0: No policy / no information available to the research team |
| R-PROMO5.3 | Does the chain restaurant commit to not use premium offers (e.g., promotional toys, games, vouchers and competitions) in marketing of products (including as part of children’s meals) other than those that meet the chain restaurants healthy standard?  *(Note: check if the company supports the EU Pledge. If yes and no other comments, then EU pledge is scored)* | 5: All forms of marketing  2.5: Some forms of marketing (e.g., excludes packaging)  0: No / no information |
| R-PROMO6 | Does the chain restaurant commit to only advertise or display ‘healthy’ sides and ‘healthy’ drinks in children’s combination meals in restaurants (for example, on menus and menu boards or in advertisements in restaurants)? | 10: Yes, commits to only advertising both healthy sides and healthy drinks for children’s meals or does not advertise children’s meals  5: Yes, commits to only advertising either healthy sides or health drinks  0: No commitment / no information available to the research team |
| R-PROMO7 | Does the chain restaurant audit/monitor its compliance with its policy on marketing to children at the?  *(Note: check if the company supports the EU Pledge. If yes and no other comments, then EU pledge is scored)* | 5: Yes, the policy is audited by an independently appointed third party  2.5: Yes, the policy independently audited  1: No, the policy is not independently audited  0: No auditing is conducted |
|  | **Classification system** |  |
| R-PROMO8.1 | What system / criteria (e.g., product classification system or nutrient profiling system) does the chain restaurant use to classify the healthiness of products for the purposes of **promotion to children and adolescents**? | 10: Adopted an official classification system (developed by WHO, PAHO, national government, etc.)  5: Developed own system that has been validated and shows strong alignment with official classification systems / dietary guidelines, published in peer-reviewed literature  2.5: Developed own system that has been validated and shows alignment with official classification systems / dietary guidelines, not published in peer-reviewed literature  0: No information / poor alignment / does not have a system |
| R-PROMO8.2 | If a proprietary product classification system has been developed, which products, nutrients and food characteristics are covered, and what are the details? | [Information only, not to be scored] |
|  | **Policy position on marketing of unhealthy foods to children** | |
| R-PROMO9.1 | Does the chain restaurant publish its policy position (in relation to government policy) on reducing the exposure of children and /or adolescents to, and the power of, the marketing of unhealthy foods? | 10: Yes, on own website  5: Yes, on industry association website  0: Not publicly available |
| R-PROMO9.2 | Does chain restaurants policy position support WHO’s position on government-led policy action related to reducing the exposure of children and adolescents to, and the power of, the marketing of unhealthy foods, as articulated in the WHO Global Action Plan for NCDs, the WHO European Food and Nutrition Action Plan 2015 - 2020 and other key WHO documents (such as the Report of the Commission on Ending Childhood Obesity)?  *According to the World Health Assembly resolution WHA63.14, marketing policy should aim to reduce the impact on children of marketing of foods high in saturated fats, trans-fatty acids, free sugars or salt by reducing both exposure of children to, and power of, marketing of foods high in these nutrients, with uniform implementation across all implementing bodies. The policy should include settings where children gather. The government should be the key stakeholder in developing the policy including implementation, monitoring and evaluation, and enforcement systems should be in place including clear definitions of sanctions. Additional details available at:*  [*http://apps.who.int/iris/bitstream/10665/44416/1/9789241500210_eng.pdf*](http://apps.who.int/iris/bitstream/10665/44416/1/9789241500210_eng.pdf)  *Part of Objective 1, EU Action plan: Establish strong measures to reduce the overall impact on children of all forms of marketing of foods high in energy, saturated fat, trans fats, sugar or salt. Experience suggests that self-regulatory, voluntary approaches have loopholes and government leadership is required.* | 10: Strong support (e.g., includes support for government-led action of marketing to children and adolescents, related to power and exposure)  5: Weak support (e.g., includes support for government-led action of marketing to children or adolescents, related to either power or exposure)  0: No details available  -5: Somewhat opposed (e.g., opposes government-led efforts to restrict some aspects of promotion to children / adolescents)  -10: Strongly opposed (e.g., opposes any actions to reduce promotion to children) |
| **R-ACCESS** | **Product accessibility** | **Maximum total points = 95** |
| R-ACCESS1 | Does the chain restaurant make a commitment to address the price / affordability of its healthy products relative to its unhealthy products? | 10: Commitment that standard prices of healthy products are lower than standard prices of comparable unhealthy products, published and applicable to Europe or multiple European countries  7.5: Commitment that standard prices of healthy products are lower than standard prices of comparable unhealthy products, not published or unclear if applicable to Europe or multiple European countries  5: Commitment that standard prices of healthy products are equivalent to standard prices of comparable unhealthy products  2.5: Broad and global commitment, not published  0: No commitment/ no information available to the research team |
| R-ACCESS2 | Does the chain restaurant have a policy that price promotions are used only on healthy products? | 10: Policy that all price promotions are for healthy products  5: Policy that factors the healthiness of products into price promotion decisions  2.5: No specific policy in the area, but ‘meal deals’ include the option of healthy sides and drinks  0: No policy / no information available to the research team |
| R-ACCESS3 | Does the chain restaurant explicitly commit to not use price incentives (such as ‘supersizing’) that incentivise consumers to purchase larger portion sizes for minimal extra cost? | 5: Yes  0: No explicit commitment / no policy information available to the research team |
| R-ACCESS4 | Does the chain restaurant commit to not provide free refills for caloric soft drinks / soda? | 5: Yes  0: No commitment / no policy information available to the research team  N/A if no free refills available |
| R- ACCESS5.1 | Does the chain restaurant have a policy that ‘assigned’ or ‘default’ drink items included as part of *children’s combination meals* are healthy items (e.g., water)?    IF APPLICABLE | 5: Yes, healthy drink items are assigned by default for all children’s combination meals  2.5: Yes, healthy drink items are available for children’s combination meals, but not by default  0: No commitment/ no policy information available to the research team |
| R-ACCESS5.2 | Does the chain restaurant have a policy that ‘assigned’ or ‘default’ drink items included as part of *‘non-children’s’ combination meals* are healthy items (e.g., water)?  IF APPLICABLE | 5: Yes, healthy drink items are assigned by default for all non-children’s combination meals  2.5: Yes, healthy drink items are available for non-children’s combination meals, but not by default  0: No commitment/ no policy information available to the research team |
| R-ACCESS6.1 | Does the chain restaurant have a policy that ‘assigned’ or ‘default’ side items included as part of *children’s combination meals* are healthy items (e.g., salad, fruit, vegetables)?  IF APPLICABLE | 5: Yes, for all children’s meals  2.5: Children’s combination meals offer healthy side items as one of the options, but not by default  0: No commitment/ no policy information available to the research team |
| R-ACCESS6.2 | Does the chain restaurant have a policy that ‘assigned’ or ‘default’ side items included as part of *‘non-children’s’ combination meals* are healthy items (e.g., salad, vegetables)?  IF APPLICABLE | 5: Yes, for all other meals (non-children’s meals)  2.5: Yes, offer ‘healthier’ options, but not by default  0: No commitment/ no policy information available to the research team |
| R-ACCESS7 | Does the chain restaurant commit to not opening new stores near schools? | (Can be multiple)  5: Commits to not opening a new location near (e.g., within 500m) of primary schools, published (2.5 if not published)  5: Commits to not opening a new location near (e.g., within 500m) of secondary schools, published (2.5 if not published) |
| R- ACCESS8 | Does the restaurant have salt sachets / shakers available only upon request? Is extra salt only available on request? | 5: Yes  0: No |
|  | **Classification system** |  |
| R-ACCESS9.1 | What system / criteria (e.g., product classification system or nutrient profiling system) does the chain restaurant use to classify the healthiness of products for the purposes of **product pricing, availability and selection**? | 10: Adopted an official classification system (developed by WHO, PAHO, national government, etc.)  5: Developed own system that has been validated and shows strong alignment with official classification systems / dietary guidelines, published in peer-reviewed literature  2.5: Developed own system that has been validated and shows alignment with official classification systems / dietary guidelines, not published in peer-reviewed literature  0: No information / poor alignment / does not have a system |
| R-ACCESS9.2 | If a proprietary product classification system has been developed, which products, nutrients and food characteristics are covered, and what are the details? | [Information only, not to be scored] |
|  | **Policy position in relation to fiscal policies** | |
| R-ACCESS10.1 | Does the chain restaurant publish its policy position (in relation to government policy) on fiscal policies to make healthier foods relatively cheaper and unhealthy foods relatively more expensive? | 10: Yes, on own website  5: Yes, on industry association website  0: Not publicly available |
| R-ACCESS10.2 | Does the chain restaurants policy position support WHO’s position on fiscal policies to make healthier foods relatively cheaper and unhealthy foods relatively more expensive, as articulated in the WHO Global Action Plan for NCDs, the WHO European Food and Nutrition Action Plan 2015 - 2020 and other key WHO documents (such as the Report of the Commission on Ending Childhood Obesity, Recommendation 1.2)?  *(ECHO Statement on Recommendation 1.2: Implement an effective tax on sugar-sweetened beverages.)*  *(Global Action Plan: consider economic tools that are justified by evidence, and may include taxes and subsidies, that create incentives for behaviours associated with improved health outcomes, improve the affordability and encourage consumption of healthier food products and discourage the consumption of less healthy options.)*  *(Part of Objective 1 EU Action plan: Consider the range of economic tools, including supply chain incentives, targeted subsidies and taxes, that could decrease or increase price, notably at point of purchase, and that could improve the affordability of a healthy diet and discourage the consumption of food products high in energy, saturated fats, trans fats, sugar or salt.)* | 10: Strong support (e.g., includes support for taxes on unhealthy foods, broadly defined, as well as subsidies for healthy foods)  5: Weak support (e.g., includes support for taxes on unhealthy foods, narrowly defined, or subsidies for healthy foods)  0: No details available  -5: Somewhat opposed (e.g., opposes taxes on unhealthy foods or subsidies for healthy foods)  -10: Strongly opposed (e.g., opposes all measures in this area) |
|  | | |
| **R-RELAT** | **Relationships with other organisations** | **Maximum total points = 90** |
| R-RELAT1 | Does the chain restaurant publish details of the **professional organisations** (e.g., professional associations for nutrition or dietetics, physical activity or exercise organisations, medical organisations or societies, etc.) and/or scientific events (e.g., conferences) it funds or supports, including awards/prizes, making clear the nature of that support? | 10: Yes, information on European activity or activity in multiple European countries is publicly available (website or document) in a consolidated and cumulative form OR active declaration/policy stating no activity in this area (either publicly available or disclosed to INFORMAS team)  5: Yes, information is available, but is not consolidated and easy to locate OR information is available at the global level only OR comprehensive information about their activities in the area provided to the project team  0: No information available / provided |
| R-RELAT2 | Does the chain restaurant publish details of the **external research** (e.g., conducted by individuals/groups/organisations) it funds or supports, including awards/prizes? | 10: Yes, information on European activity or activity in multiple European countries is publicly available (website or document) in a consolidated and cumulative form OR active declaration/policy stating no activity in this area (either publicly available or disclosed to INFORMAS team)  5: Yes, information is available, but is not consolidated and easy to locate OR information is available at the global level only OR comprehensive information about their activities in the area provided to the project team  0: No information available / provided |
| R-RELAT3 | **For philanthropic funding,** does the chain restaurant publish details of the groups or organisations it funds or supports? | 10: Yes, information on European activity or activity in multiple European countries is publicly available (website or document) in a consolidated and cumulative form OR active declaration/policy stating no activity in this area (either publicly available or disclosed to INFORMAS team)  5: Yes, information is available, but is not consolidated and easy to locate OR information is available at the global level only OR comprehensive information about their activities in the area provided to the project team  0: No information available / provided |
| R-RELAT4.1 | Does the chain restaurant publish details of the **nutrition education / healthy diet oriented programs** it funds or supports? | 10: Yes, information on European activity or activity in multiple European countries is publicly available (website or document) in a consolidated and cumulative form OR active declaration/policy stating no activity in this area (either publicly available or disclosed to INFORMAS team)  5: Yes, information is available, but is not consolidated and easy to locate OR information is available at the global level only OR comprehensive information about their activities in the area provided to the project team  0: No information available / provided |
| R-RELAT4.2 | **For nutrition education / health diet oriented programs**, does the chain restaurant have a commitment to align programs to national or regional dietary guidelines? | [Information only, not to be scored] |
| R-RELAT5 | Does the chain restaurant publish details of the **active lifestyle programs** (sports, physical activity) it funds or supports? | 10: Yes, information on European activity or activity in multiple European countries is publicly available (website or document) in a consolidated and cumulative form OR active declaration/policy stating no activity in this area (either publicly available or disclosed to INFORMAS team)  5: Yes, information is available, but is not consolidated and easy to locate OR information is available at the global level only OR comprehensive information about their activities in the area provided to the project team  0: No information available / provided |
| R-RELAT6 | Does the chain restaurant publish details of its involvement in public-private partnerships and/or joint ventures with government organisations / agencies? (in addition to those covered as part of R-RELAT4.1 and R-RELAT5) | 10: Yes, information on European activity or activity in multiple European countries is publicly available (website or document) in a consolidated and cumulative form OR active declaration/policy stating no activity in this area (either publicly available or disclosed to INFORMAS team)  5: Yes, information is available, but is not consolidated and easy to locate OR information is available at the global level only OR comprehensive information about their activities in the area provided to the project team  0: No information available / provided |
| R-RELAT7 | Does the chain restaurant publish details of its political donations?  (when not prohibited by government policy) | 10: Yes, information on European-level activity is publicly available (on a company website or document) OR declaration of no activity in this area  5: Yes, for specific European countries only OR actively declares no activity in some countries  0: No |
| R-RELAT8 | Does the chain restaurant publish its membership / support for / ownership of industry associations, think tanks, interest groups, community organisations or other organisations that lobby in relation to population nutrition and/or obesity and NCD issues? | 10: Yes, information on European activity or activity in multiple European countries is publicly available (website or document) in a consolidated and cumulative form OR active declaration/policy stating no activity in this area (either publicly available or disclosed to INFORMAS team)  5: Yes, information is available, but is not consolidated and easy to locate OR information is available at the global level only OR comprehensive information about their activities in the area provided to the project team  0: No information available / provided |
| R-RELAT9 | Does the company make publicly available its submissions (or submissions with which the company is associated, such as through industry associations) to public consultations regarding relevant population nutrition policies? | 10: Yes, on company website or in a document that is publicly available or available upon request OR active declaration/policy stating no activity in this area (either publicly available or disclosed to INFORMAS team)  5: Yes, through industry association/European website/document  0: No |
| R-RELAT10 | Does the chain restaurant have written policy and guidelines related to any of the above (funding or support for professional organisations, external research, philanthropic funding, nutrition education / healthy diet oriented programs, active lifestyle programs), including details of how it will be involved in these activities? | [Information only, not to be scored] |
